# Controlling the Deposition Process of Nanoarchitectonic Nanocomposites Based on {Nb_6−x_Ta_x_X^i^_12_}^n+^ Octahedral Cluster-Based Building Blocks (X^i^ = Cl, Br; 0 ≤ x ≤ 6, n = 2, 3, 4) for UV-NIR Blockers Coating Applications

**DOI:** 10.3390/nano12122052

**Published:** 2022-06-15

**Authors:** Clément Lebastard, Maxence Wilmet, Stéphane Cordier, Clothilde Comby-Zerbino, Luke MacAleese, Philippe Dugourd, Toru Hara, Naoki Ohashi, Tetsuo Uchikoshi, Fabien Grasset

**Affiliations:** 1 Univ Rennes, CNRS, ISCR, UMR6226, F-35000 Rennes, France; maxence.wilmet@saint-gobain.com (M.W.); stephane.cordier@univ-rennes1.fr (S.C.); 2 CNRS-Saint Gobain-NIMS, IRL3629, Laboratory for Innovative Key Materials and Structures (LINK), National Institute for Materials Science (NIMS), 1-1 Namiki, Tsukuba 305-0044, Japan; ohashi.naoki@nims.go.jp (N.O.); uchikoshi.tetsuo@nims.go.jp (T.U.); 3 Saint Gobain Research Paris, F-93300 Aubervilliers, France; 4 Univ Lyon, Univ Claude Bernard Lyon 1, CNRS, Institut Lumière Matière, F-69622 Lyon, France; clothilde.comby-zerbino@univ-lyon1.fr (C.C.-Z.); luke.mac-aleese@univ-lyon1.fr (L.M.); philippe.dugourd@univ-lyon1.fr (P.D.); 5 Research Center for Structural Materials, NIMS, 1-2-1 Sengen, Tsukuba 305-0047, Japan; hara.toru@nims.go.jp; 6 Research Center for Functional Materials, NIMS, 1-1 Namiki, Tsukuba 305-0044, Japan

**Keywords:** octahedral metal clusters, thin films, nanocomposites, UV and NIR blocker, solar glazing

## Abstract

The antagonism between global energy needs and the obligation to slow global warming is a current challenge. In order to ensure sufficient thermal comfort, the automotive, housing and agricultural building sectors are major energy consumers. Solar control materials and more particularly, selective glazing are part of the solutions proposed to reduce global energy consumption and tackle global warming. In this context, these works are focused on developing new highly ultraviolet (UV) and near-infrared (NIR) absorbent nanocomposite coatings based on K_4_[{Nb_6-x_Ta_x_X^i^_12_}X^a^_6_]. (X = Cl, Br, 0 ≤ x ≤ 6) transition metal cluster compounds. These compounds contain cluster-based active species that are characterized by their strong absorption of UV and NIR radiations as well as their good transparency in the visible range, which makes them particularly attractive for window applications. Their integration, by solution processes, into a silica-polyethylene glycol or polyvinylpyrrolidone matrices is discussed. Of particular interest is the control and the tuning of their optical properties during the integration and shaping processes. The properties of the solutions and films were investigated by complementary techniques (UV-Vis-NIR spectrometry, ESI-MS, SEM, HRTEM, etc.). Results of these works have led to the development of versatile solar control coatings whose optical properties are competitive with commercialized material.

## 1. Introduction

**Figure 1:**
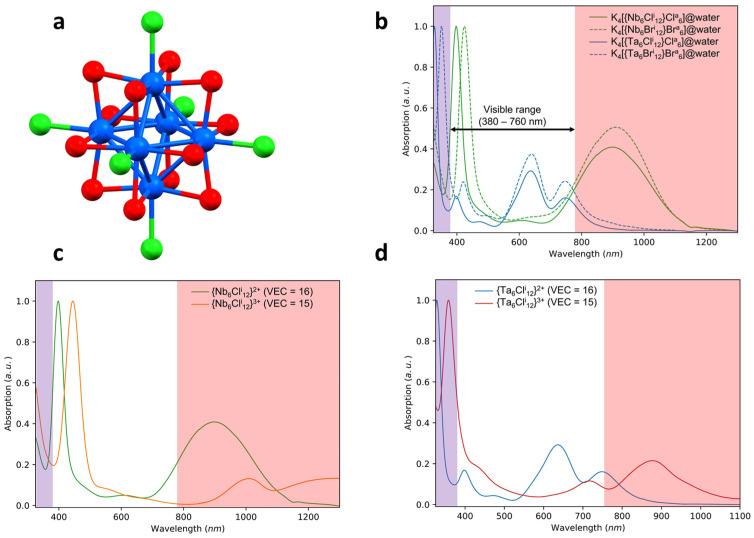
(**a**) Structural view of octahedral [{M_6_X^i^_12_}X^a^_6_]^n−/+^ cluster unit (Blue = M; red = X^i^; green = X^a^). (**b**–**d**) Normalized UV-Vis-NIR absorption spectra of the homometallic cluster compounds. The solubility of these compounds varies according to the nature of the metal and the ligands, so, the UV-Vis-NIR absorption spectra were normalized based on the most intense absorption band between 300 and 500 nm.

Nanocluster compounds with the general formula K_4_[{Nb_6−x_Ta_x_X^i^_12_}X^a^_6_]. (X = Cl, Br; 0 ≤ x ≤ 6) constitute a part of the large family of the octahedral transition metal atom clusters defined by Cotton [1]. These metal atom clusters are based on anionic cluster units [{Nb_6−x_Ta_x_X^i^_12_}X^a^_6_]^4−^ which are nanosized molecules containing an octahedral cluster. The 6 metal atoms of the cluster are connected by direct metal–metal bonds and linked with 12 inner ligands (X^i^, edge-bridging positions) and 6 apical ligands (X^a^, terminal position) (Figure 1) [2]. These [{Nb_6−x_Ta_x_X^i^_12_}X^a^_6_]^4−^ nanosized molecules or nanocluster units combine with counter cations (K in this specific case) in order to form crystallized cluster compounds. In the Sixties, the first syntheses started with the compound K_4_[{Nb_6_Cl^i^_12_}Cl^a^_6_]. (x = 0) by using, for instance, the precursors Nb_3_Cl_8_ or Nb_6_Cl_14_ and KCl, a reducing atmosphere and a high synthesis temperature (800 °C) or a long synthesis time (4 days) [3,4]. Alternative syntheses using metal as reducer agent for the compositions with x = 0 or 6 were proposed by Fleming et al., then by Koknat et al., and very recently by Wilmet et al., in order to reduce the number and to optimize the amount of the starting precursors and/or to simplify the experimental protocol of the reaction [3,5,6,7]. These popular octahedral cluster compounds exhibit physicochemical behaviors of great interest, such as a strong redox activity in combination with strong ultraviolet-visible-near infrared (UV-Vis-NIR) absorption properties to afford green to red–brownish solutions [8,9,10]. Recently, these specific properties were used to develop new UV and NIR absorbent nanocomposites [11,12,13,14,15]. Moreover, some tantalum cluster compounds have been known for decades as radiographic contrast agents [16], as a commercial tool by Jena Biosciences for phase determination of large biological assemblies by X-ray crystallography [17,18,19]. In order to extend these properties, the first compounds synthesized with x ≠ 0 or 6 were prepared by W. Preetz and K. Harder for the series Na_4_[{Nb_6−x_Ta_x_Cl^i^_12_}Cl^a^_6_]. (1 ≤ x ≤ 5) [20]. The protocol developed by these authors consists of the reduction of a niobium or tantalum halide by tantalum or niobium, respectively, in the presence of sodium chloride. The different units are isolated by ion exchange chromatography using a positively charged resin, but no crystallographic data have been reported for these systems. Nevertheless, the vibrational bands of these heterometallic cluster compounds evolve with the value of x, without being a superposition of the homometallic pattern bands, which is a good indicator for potential tunability of the properties. In this work, the K_4_[{Nb_6−x_Ta_x_X^i^_12_}X^a^_6_]. (X = Cl, Br; 0 ≤ x ≤ 6) series cluster compounds were prepared by solid-state chemistry, at a large scale, using an optimized method derived from that of F. W. Koknat et al., and Wilmet et al., for x = 6 compounds [5,6,7].

The main purpose of this new study is to investigate the behavior of the [{Nb_6-x_Ta_x_X^i^_12_}X^a^_6_]^4−^ cluster units as building blocks for preparing tunable nanocomposites coatings with optimum optical properties for UV and NIR absorption. Two types of matrixes were selected, polyvinylpyrrolidone (PVP) or silica-polyethylene glycol (SiO_2_-PEG), to disperse the K_4_[{Nb_6-x_Ta_x_X^i^_12_}X^a^_6_] cluster compounds. The multifunctional nanocomposites with highly controlled dispersion of the nanoclusters have been prepared by very simple, nontoxic and low-cost solution chemistries. The resulting solutions have been used to fabricate highly transparent in visible and UV and NIR absorbent coatings by Mayer bar coating or drop-casting. The properties of the films were investigated by complementary techniques (UV-Vis-NIR spectrometry, Raman, ESI-MS, HRTEM, etc.). We will demonstrate that the UV and NIR absorption of such a system is strongly dependent on the amount of the cluster units and their oxidation state into the nanocomposite. The role played by the ligand X is also important, and we will demonstrate that Cl-based compounds present better optical properties for their reduced forms, whereas the oxidized form of the Br-based compounds is more interesting for the targeted application. By varying and controlling these parameters, a large variety of colors and UV and NIR absorption can be achieved, as well as very interesting values of the figure-of-merit (FOM) T_L_, T_E_, S_NIR,_ haze and clarity (see supplementary information for details) for solar glazing applications.

## 2. Materials and Methods

The K_4_[{Nb_6−x_Ta_x_X^i^_12_}X^a^_6_] (X = Cl, Br; 0 ≤ x ≤ 6) cluster compounds were prepared by solid-state chemistry using an optimized method derived from that of F. W. Koknat et al., and Wilmet et al. [5,6,7]. Potassium chloride (KCl; Alfa Aesar—99%, Haverhill, MA, USA), potassium bromide (KBr; Acros—99%, Waltham, MA, USA), niobium pentachloride (NbCl_5_; Strem—99.99%, Newburyport, MA, USA), niobium pentabromide (NbBr_5_; Strem—99.9%), tantalum pentachloride (TaCl_5_; Alfa Aesar—99.8%), tantalum pentabromide (TaBr_5_; Alfa—99.9%), niobium powder (Alfa Aesar—99.8%) and tantalum powder (Alfa Aesar—99.97%) were used as starting precursors.

The precursors are weighted and mixed in an agate mortar in a glove box under a controlled argon atmosphere according to the following chemical reaction:

$$4\mathrm{KX}+\frac{14}{5}\;(\frac{6-x}{6}){\mathrm{NbX}}_{5}+\frac{14}{5}\;(\frac{x}{6}){\mathrm{TaX}}_{5}\;+7.5(\frac{6-x}{6})\mathrm{Nb}+4(\frac{x}{6})\mathrm{Ta}\;\to \;K_{4}[\{{\mathrm{Nb}}_{6-x}{\mathrm{Ta}}_{x}{X^{i}}_{12}\}{X^{a}}_{6}]+4.3(\frac{6-x}{6})\mathrm{Nb}+0.8(\frac{x}{6})\mathrm{Ta}$$

Then, the homogenized powder is introduced into a silica tube which is vacuum sealed before being introduced into a tube furnace to undergo heat treatment. The tube undergoes a temperature rise of 1 °C.min^−1^ from room temperature to 600 °C. This temperature is maintained for 24 h before cooling by thermal inertia to ambient temperature. The tube is finally opened in a glove box, in order to avoid oxidation and/or hydrolysis of the cluster compounds. As already demonstrated for Ta_6_ cluster compounds, just before the preparation of the solutions, a large part of the potassium salt and metal impurities are removed by suitable dissolution in acetone followed by filtrations (0.2 μm) or by the syntheses of aquo-complexes [7]. The characterizations of the cluster compounds are available in these references: [7,21,22].

PVP (Sigma-Aldrich, St. Louis, MO, USA), TEOS (99% Merck, Darmstadt, Germany), PEG (Aldrich (400 g∙mol^−1^, 1450 g∙mol^−1^ and 10,000 g∙mol^−1^) or Acros (600 g∙mol^−1^)) were used as starting materials for the synthesis of the matrixes without further purification.

The integration of the K_4_[{Nb_6-x_Ta_x_X^i^_12_}X^a^_6_] cluster compounds in the SiO_2_-PEG matrix is carried out according to a protocol derived from the synthesis of the SiO_2_-PEG matrix alone [23,24,25]. For a total volume of approximately 20 mL, 200 mg of cluster compounds are dissolved in the acidified (pH = 2 to 7, HCl, 37%_wt_) water–ethanol mixture (4.84 g/3.09 g, molar ratio 4/1). The solution is then placed under magnetic stirring for 24 h, then filtered (0.2 μm). PEG and TEOS (7 g) are added successively for the synthesis of the matrix. The molar proportion of PEG in the silica, as well as the length of the chain, has been changed, respectively, between 1%_mol_ and 30%_mol_ and between 400 g∙mol^−1^ and 10,000 g∙mol^−1^. The solution is then placed under magnetic stirring for 10 h for homogenization. The best properties were obtained for PEG with a molar mass of 600 g∙mol^−1^ and a %_mol_ = 10. The solutions are stable from 2 days to several weeks depending on the pH conditions (see Table S1). Before the deposition, the best solutions were concentrated by using an evaporator, and around one third of the solvent was removed until the final viscosity reached 20 cP. SiO_2_-Polydimethylsiloxane and SiO_2_ were tested as matrixes, but the results were poor compared to SiO_2_-PEG [21]

The colloidal solutions containing PVP were prepared by dissolving an appropriate amount of cluster compounds (denoted Y in the following and ranging from 1 to 20 g∙L^−1^) in water solution. After a filtration step, in order to get rid of metallic and non-soluble impurities, PVP was dissolved and maintained under magnetic stirring until homogenization of the solution. Different concentrations and molar masses of PVP (from 5 to 50%_wt_, 10,000 g∙mol^−1^, 40,000 g∙mol^−1^, 1,300,000 g∙mol^−1^) were tested to get a compromise between viscosity, thickness and cluster concentration. The best results were obtained for PVP with a molar mass of 1,300,000 g∙mol^−1^ and %_wt_ = 10. All the solutions were stable for several months.

From these stable colloidal solutions, the films with PVP as matrix (noted @PVP) were deposited on soda lime glass slides (up to 10 cm × 15 cm) by drop-casting, and a homemade upgraded Mayer bar coater was used for the films with SiO_2_-PEG (noted @SiO_2_-PEG) (see Figure S5). The formula of the corresponding films is noted as {Nb_6-x_Ta_x_X^i^_12_}@SiO_2_-PEG or {Nb_6−x_Ta_x_X^i^_12_}-Y@PVP. All the films were dried at room temperature for 24 h. Some films were annealed in air at 50 °C, 80 °C and 100 °C for several hours.

Ultraviolet-visible spectroscopy (UV-Visible-NIR) was performed in a spectrophotometer (V7770, Jasco, Oklahoma City, OK, USA), with solutions taken in absorbance mode with the same dilution and with films taken in transmission mode in the range 200–2500 nm. A HazeGard Plus hazemeter apparatus from OAKLAND Inst. Corp. (Shakopee, MN, USA) was used to measure at once the haze, transmission and clarity values following the Standard Test Method “ASTM D-1003”.

Mass spectrometry (ESI-MS) measurements with ionization by electrospray or nanospray source were recorded on a quadrupole time-of-flight mass spectrometer (microtof-Q, Bruker-Daltonics, Bremen, Germany). The samples were analyzed both in negative and positive ion mode. Each solution sample was prepared to approximately 50 μmol∙L^−1^ (residual impurities preventing one to attain a completely quantitative concentration) in the following different solvents: water, ethanol and acetone. The water and ethanol solutions were infused directly in an electrospray source using a syringe pump (flow rate 180 μL∙h^−1^) and the ESI process was assisted with a dry gas at 80 °C. The acetone solutions were infused directly in a nanospray source with dry gas temperature set at 55 °C.

Raman scattering spectra were acquired on powders and thin films at room temperature using a LabRamHigh resolution spectrometer coupled with a confocal microscope (Horiba Jobin Yvon, Edison, NJ, USA), 600 g/mm gratings and 10× objective. A He-Ne 633 nm laser was used for scattering excitation. Raman spectra were recorded at room temperature with 100 s exposition and 2 accumulations. The calibration of the Raman spectrometer was performed using the main Raman band of silicon wafer (520 cm^−1^). A soda–lime glass sample holder was used in all cases.

In order to investigate the dispersion of the nanocluster directly, TEM observation was carried out. TEM samples were pick-upped and thinned using a FIB-SEM instrument (ZEISS Auriga Laser) with an accelerated voltage of 30 kV (gallium ion). The beam current for final thinning was 50 pA, which was low enough to avoid introducing damage. TEM observation was performed with a Scanning-TEM (STEM) mode operating at 200 kV. The TEM used was JEOL JEM-2800 (JEOL Ltd., Tokyo, Japan). Because this sample was very sensitive for the electron beam, image recording should had been done in a short time. Under the operated condition for these observations, no morphological change due to the beam damage was observed.

## 3. Results and Discussion

### 3.1. Study of the Powders and Solutions

**Figure 2:**
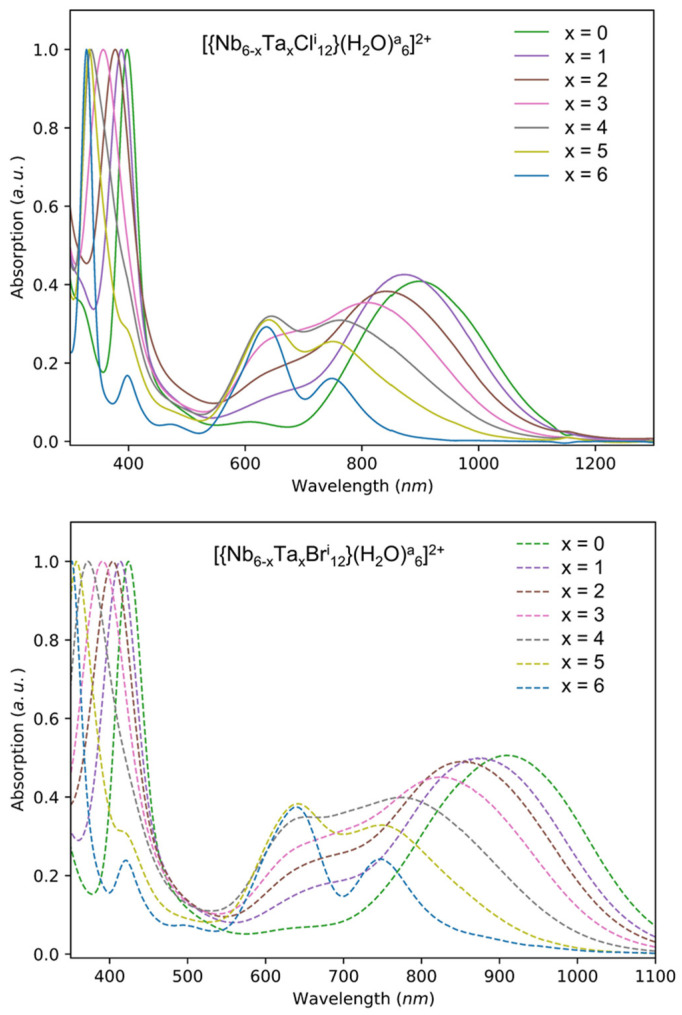
Normalized UV-Vis-NIR absorption spectra of the heterometallic K_4_{Nb_6−x_Ta_x_X^i^_12_}(H_2_O)^a^_6_] cluster compounds (X = Cl, Br; 0 ≤ x ≤ 6; VEC = 16) dispersed in water.

It is well known that K_4_[{Ta_6_X^i^_12_}X^a^_6_] and K_4_[{Nb_6_X^i^_12_}X^a^_6_] compounds, even prepared by solid-state chemistry at high temperature, are highly soluble in common solvents (water, alcohols, ketones, etc.), affording interesting colored metal complexes with redox activities. The different solutions of these clusters exhibit specific ultraviolet, visible and/or near infrared absorption (or transmission) properties. For instance, the green color of the Ta_6_ based-cluster units in solution and in particular, the deep emerald-green color of the [{Ta_6_X^i^_12_}X^a^_6_]^4−^ (X = Cl, Br) cluster core has been known for more than one century [23,24,25]. Moreover, in solution, these cluster units can be oxidized (with possible reversible processes) and/or the apical ligands can be exchanged, leading to drastic modulations of their optical properties [8,9,11]. For instance, the dispersion in acetone results in a solution whose color depends on the oxygen atmosphere. Under an inert atmosphere (deoxygenated) using Schlenk tube techniques, the solution exhibits a stable emerald-green color characteristic of the reduced tantalum nanoclusters, whereas the solution turns immediately to red-brown when exposed to air by the oxidation of the nanoclusters. Electronically, this changing of color corresponds to a decrease of the valence electron concentration (VEC) per cluster from 16 to 15 or 14 [26,27,28,29]. The emerald-green solution is characteristic of the presence of [{Ta_6_Br^i^_12_}X^a^_6_]^4−^ cluster unit species (VEC = 16) whereas yellow and red-brown colors were mostly referenced for the [{Ta_6_Br^i^_12_}X^a^_6_]^3−^ (VEC = 15) and [{Ta_6_Br^i^_12_}X^a^_6_]^2−^ species (VEC = 14), respectively [10]. In parallel, in water, it was demonstrated that the 6 apical bromine ligands are exchanged by 6 molecules of H_2_O to give the [{Ta_6_Br^i^_12_}(H_2_O)^a^_6_]^4−^ cluster unit without VEC changing [7]. To resume, all the previous works have highlighted the existing relationships between the structure of the homometallic cluster and the resulting optical properties. Various effects have been observed and are mainly related to the nature of the metal and the ligands and to the degree of oxidation of the cluster units. These effects can be controlled mainly by the choice of the solvent in which the cluster compound is dissolved and the handling conditions (pH, oxidizing/reducing medium, O_2_, etc.). For a better understanding, Figure 1b reports the well-known absorption spectra for the K_4_[{Nb_6_X^i^_12_}X^a^_6_]. (x = 0) and K_4_[{Ta_6_X^i^_12_}X^a^_6_]. (x = 6) cluster compounds (X = Cl, Br) dispersed in water with a VEC value of 16, i.e., for the reduced form. The Figure 1c,d report the same clusters with different VEC values, i.e., 16 or 15, measured in water or oxygenated acetone [22]. The fine signature of the spectra of these homometallic clusters mainly depends on the composition of the cluster: either Cl or Br ligands, Nb_6_ or Ta_6_ metals and the VEC values (either 16 or 15). Indeed, the role played by the ligands Cl or Br is important (by keeping constant the metal and VEC = 16), as observed in the Figure 1a. The high energy part of the spectra for Br-based metal atom cluster units are always red-shifted compared to the Cl-based metal atom clusters units, which could impact the color of the solution, especially for the Nb_6_ cluster compounds. Moreover, on Figure 1b–d, we can notice that for a VEC = 16, the associated spectra present a fine and intense absorption band located in the UV (M = Ta) or the beginning of the visible (M = Nb) range. The influence of the ligands on the lower energy part of the spectra is very low; one (M = Nb) or two (M = Ta) bands are always observed at the same position in the red and/or NIR range, respectively, no matter the ligands. Regarding the role played by the metal, it is clear that Nb_6_ cluster units (by keeping constant the VEC = 16) present a stronger absorption in the NIR than the Ta_6_ cluster units. For a VEC = 15 (Figure 1c,d), the spectra recorded after the dissolution in acetone are characterized by a shift at higher wavelengths which corresponds to the appearance of new bands (and the disappearance of those observed on the spectra of the same compound dissolved in water) consistent with oxidation of cluster units (by keeping constant the metal and ligands) [7,10]. All these results demonstrate that Cl-based compounds present better optical properties for the targeted application at their reduced forms, whereas the oxidized forms of the Br-based compounds seem to be more interesting. In order to tune and optimize the optical properties of the nanocomposites, we focused on the compositions of the heterometallic K_4_[{Nb_6-x_Ta_x_X^i^_12_}X^a^_6_] cluster compounds (X = Cl, Br; 0 ≤ x ≤ 5). The objective is to obtain cluster compounds capable of combining the best properties of absorption in the NIR of the Nb_6_ clusters and those of absorption in the UV of the Ta_6_ clusters. This objective must be achieved while increasing concomitantly the transmission in the visible spectrum, as shown in Figure 1b. The properties of the heterometallic clusters must be controlled from their synthesis to their integration into the matrixes. The preliminary results obtained by UV-Vis-NIR and by mass spectrometry (ESI-MS) of the K_4_[{Nb_6-x_Ta_x_X^i^_12_}X^a^_6_] cluster compounds dispersed in water are presented in the Figure 2, Figures S1–S3 and the Table S1. The UV-Visible-NIR absorption spectra of the K_4_[{Nb_6-x_Ta_x_X^i^_12_}(H_2_O)^a^_6_] cluster compounds dispersed in water are reported in Figure 2.

As expected, the UV-Vis-NIR spectra of the heterometallic clusters have intermediate shapes to those observed for the homometallic clusters and fully confirm the possibility to tune the optical properties of the nanocomposites. For the ESI-MS studies, the dispersion in water showed similarities with homometallic clusters compounds (x = 0 and 6), with a total substitution of the 6 apical ligands by H_2_O molecules and no signal in negative mode [7,21,22,30]. The dissolution of K_4_[{Nb_6-x_Ta_x_X^i^_12_}X^a^_6_] cluster compounds (X = Cl, Br; 1 ≤ x ≤ 5) in water leads to a solution of the stable species [{Nb_6-x_Ta_x_X^i^_12_}(H_2_O)^a^_6_]^2+^ (X = Cl, Br; 1 ≤ x ≤ 5). It should be noted that for x = 1, we detected the presence of [{Nb_5_Ta_1_X^i^_12_}(H_2_O)^a^_5_(OH)^a^_1_]^+^ (X = Cl, Br) motifs in aqueous solution and this is analogous to that of the x = 0 homometallic [21,22,30]. For x > 1, no unit containing a hydroxyl apical ligand was detected. Nevertheless, as observed in Table S2, for some solutions, several cluster units are detected with an x − 1 or x + 1 value in addition to the x targeted value. Nevertheless, the ion presenting the most intense signal is often that whose composition x was targeted during the synthesis, except for the compositions with x = 3 and 4 for the chlorinated compounds. For these two specific values, surprisingly the most intense signal corresponds to the composition unit x + 1. In the same way, the results of mass spectrometry studies for the dispersion in acetone, in negative mode, also showed similarities with the homometallic clusters compounds, i.e., an oxidation of the cluster units without apical ligand substitution as expected (Table S2) [7,21,22,30]. All the heterometallic cluster units observed in ESI-MS in acetone are of VEC = 14 ([{Nb_6-x_Ta_x_X^i^_12_}X^a^_6_]^2−^) instead of VEC = 16 ([{Nb_6-x_Ta_x_X^i^_12_}X^a^_6_]^4−^). The situation is more confusing in ethanol because [Nb_6-x_Ta_x_Cl_12_(H_2_O)_n_(OH)(EtOH)_m_]^1+^ (with n = 1, 2; m = 3, 4 and n + m = 5) or [Nb_6-x_Ta_x_Br_12_(H_2_O)_n_(EtOH)_m_]^2+^ (with n = 5, 6; m = 0,1 and n + m = 6) cluster units could be detected. The important point is that the cluster units are not oxidized in alcohol media as for water-based solutions. Based on these results, a mixture of water/ethanol (4/1) and distilled water were selected as the best solvents for SiO_2_-PEG and the PVP nanocomposites, respectively. Acetone was excluded because of the strong oxidation process in the presence of oxygen. To conclude about these MS studies, these results confirmed that synthesis by solid chemistry at high temperature leads sometimes to the concomitant formation of the compositions (x − 1), x and (x + 1); nevertheless, the composition formed mainly corresponds to the targeted value of x.

The integration of the nanoclusters and coating processes must not impact the optical properties of the nanoclusters. We will focus in particular on optimizing the processes of incorporating the cluster compounds into the matrixes in order to control their dispersion and their degree of oxidation and therefore, their optical properties (i.e., a strong absorption in the NIR (800–1100 nm)). The SiO_2_-PEG matrix is a hybrid organic/inorganic matrix obtained by a sol-gel type process and PVP is a commercial organic polymer. These matrixes meet criteria compatible with a solar application (transparency, implementation, cost, etc.) [31,32,33].

### 3.2. Study of the {Nb_6-x_Ta_x_X^i^_12_}@SiO_2_-PEG Films

**Figure 3:**
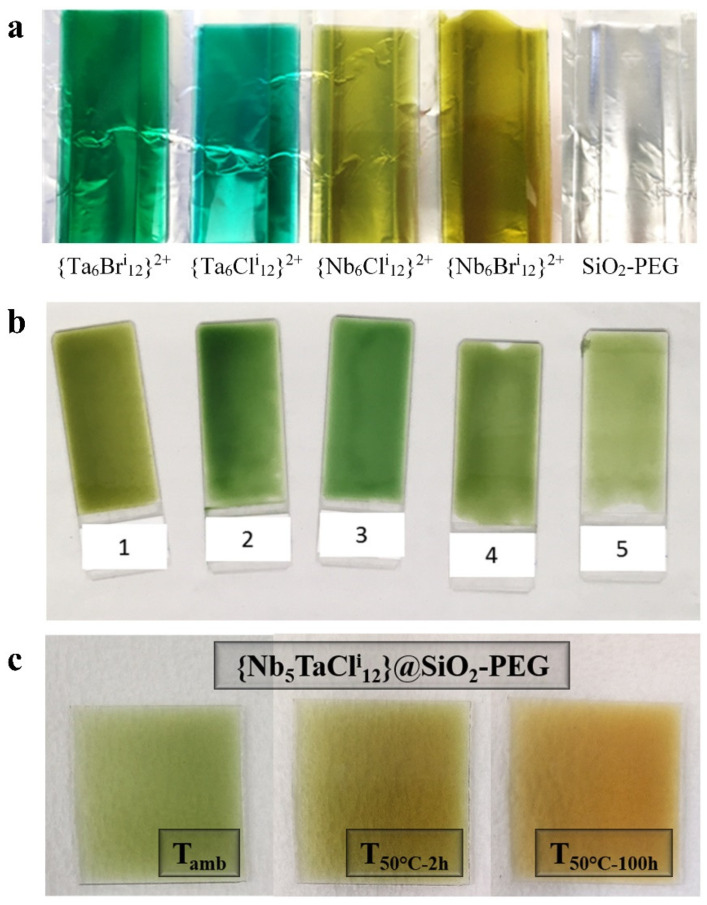
Camera images of the {Nb_6−x_TaCl^i^_12_}@SiO_2_-PEG nanocomposites thin films: (**a**,**b**) as-deposited for 0 ≤ x ≤6 (sample sizes are 7.5 cm × 2.5 cm), (**c**) {Nb_5_TaCl^i^_12_}^n +^ @SiO_2_-PEG nanocomposite films after being annealed at 50 °C for 2 h and 100 h (samples are 2.5 cm × 2.5 cm).

There are a very large number of organic–inorganic hybrid functional nanocomposite materials whose properties reach or even exceed the properties of metals, ceramics or plastics [34]. Our choice of the SiO_2_-PEG matrix is justified by the fact that this hybrid matrix is in visibility highly transparent (see Figure 3a and Figure S4) with good mechanical and adhesion properties to a glass surface. Indeed, the use of PEG polymer makes it possible to increase the elasticity, the adhesion and the hydrophilicity of the silica type matrix [35]. Moreover, the role of the PEG is therefore not only to provide flexibility and elasticity between the SiO_4_ entities but can also stabilize cations [36,37,38]. Different molar masses and concentration of PEG were tested, and the best results were obtained for PEG with M = 600 g/mol (noted PEG-600) at 10%_mol_ in silica. Indeed, beyond 1450 g∙mol^−1^, the PEG chains induce light scattering in the film if the temperature is lower than the PEG crystallization temperature. Below 2.5%_mol_, it is possible to obtain transparent films but the mechanical properties are weak. Finally, it was necessary to study the effect of the pH on this hybrid nanocomposite. It is well known that the pH modifies the hydrolysis and condensation reaction rates and at the end, modifies the structure of the polymers formed. Moreover, previous studies on Nb_6_ or Ta_6_ metal atom clusters indicated a strong correlation between the pH value and the VEC values of the clusters [7,30]. Several solutions containing the water–ethanol mixture have been prepared and the pH was adjusted between 2 and 7 by adding acid (hydrochloric acid, nitric acid, acetic acid or oxalic acid). The PEG (600 g∙mol^−1^) is added at 10 mol% then the TEOS, given, initially, biphasic and heterogeneous solutions. It is therefore stirred at 350 rpm until complete homogenization. Table S1 summarizes the times required before the hydrolysis and condensation reactions by using hydrochloric acid. The time indicated for hydrolysis corresponds to the moment when the solutions become homogeneous, and for condensation, it corresponds to the moment when the solutions become a gel. In order to have the higher hydrolysis and lower condensation, the best pH was fixed between 2 and 3. To resume, the matrix that was used in the following results therefore contains a quantity of PEG (600 g∙mol^−1^) at 10 mol% for a pH between 2 and 3. Each member of the full family of the K_4_[{Nb_6−x_Ta_x_X^i^_12_}X^a^_6_] cluster compounds (X = Cl, Br; 0 ≤ x ≤ 6), including homometallic and heterometallic nanoclusters, was dispersed into the SiO_2_-PEG matrix. The {Nb_6−x_Ta_x_X^i^_12_}@SiO_2_-PEG films were prepared, using the concentrated SiO_2_-PEG solutions, by drop-casting or homemade Mayer-bare coating up to 10 cm × 15 cm substrates (see Figure S5) (dip- and spin-coated were tested without better results). The camera images of the films (2.5 cm × 7.5 cm) with the homometallic {M_6_X^i^_12_}^2+^ cluster cores (M = Nb, Ta; X = Cl, Br (x = 0 and 6), VEC = 16) and the film with the heterometallic {Nb_6-x_Ta_x_Cl^i^_12_}^2+^ cluster cores (1 ≤ x ≤ 5, VEC = 16) are presented in Figure 3a,b respectively. The results obtained with the bromine compounds are presented in Figure S6. As an example, Figure S7 shows the UV-Visible-NIR spectra of the {Nb_6−x_Ta_x_X^i^_12_}@SiO_2_-PEG films based on the {Nb_6-x_Ta_x_Cl^i^_12_}^2+^ cluster cores (0 ≤ x ≤ 6; VEC = 16).

As observed in Figure 3a,b, Figures S6 and S7, the as-deposited films show a high transparency in terms of visibility as well as intense colorations when they contain cluster units. Even if the nanocomposite films obtained seem relatively homogeneous in terms of thickness and cluster concentration, we evaluated and quantified the microstructure by different imaging techniques being carried out: optical microscope, scanning electron microscopy and transmission electron microscopy. The thickness of the films is measured using an optical microscope at the interface between the substrate and the film, as shown in Figure S8. The thickness of the films can vary from 10 to 250 μm depending on the deposition techniques and the volume of the solution poured onto the surface of the substrate. Although UV-Vis-NIR observations did not show any notable differences, an effort to study the homogeneity of the clustered units in the film was performed by SEM observations coupled with energy dispersive spectroscopy (EDS). The SEM image and the EDS analysis are shown in Figure S9. The dispersion seems to be homogeneous at a large scale.

**Figure 4:**
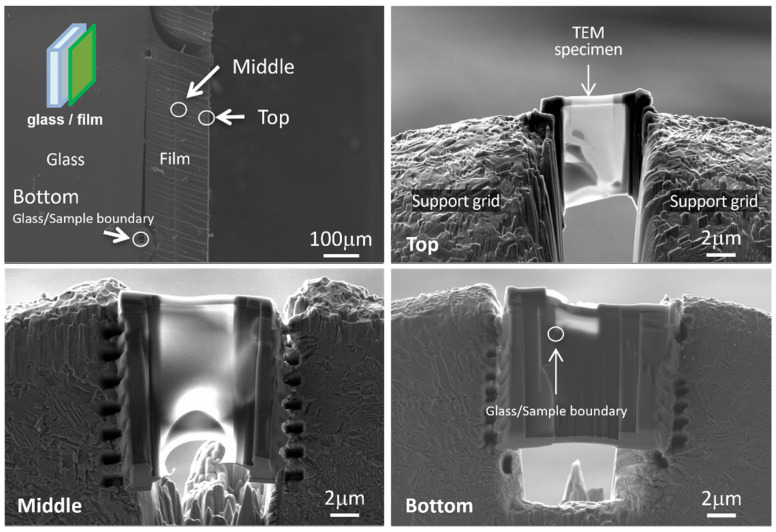
SEM images of the samples prepared by FIB.

**Figure 5:**
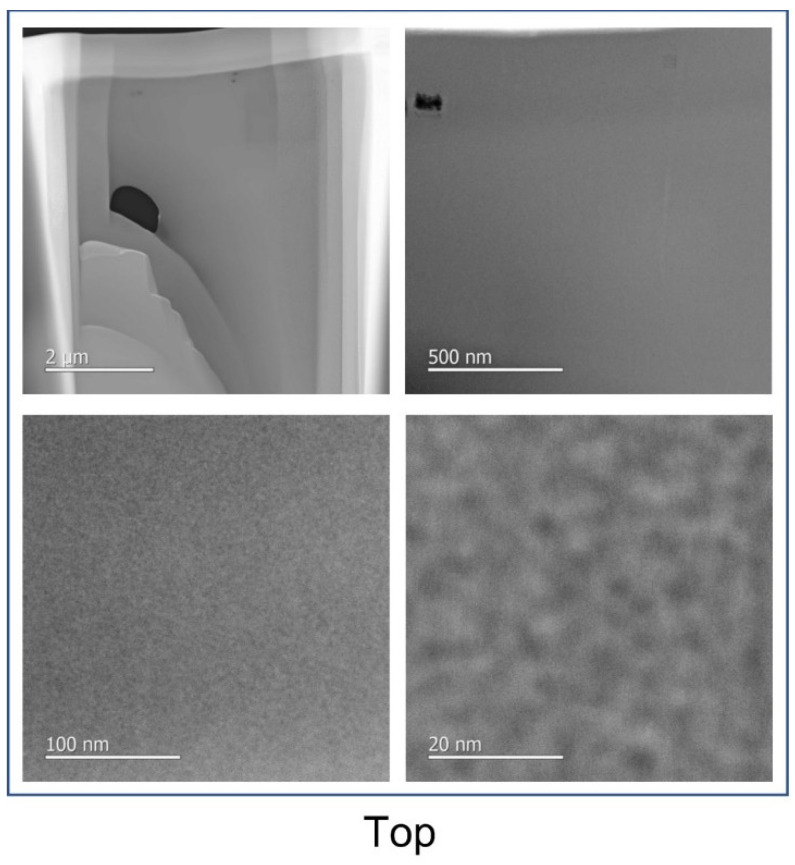

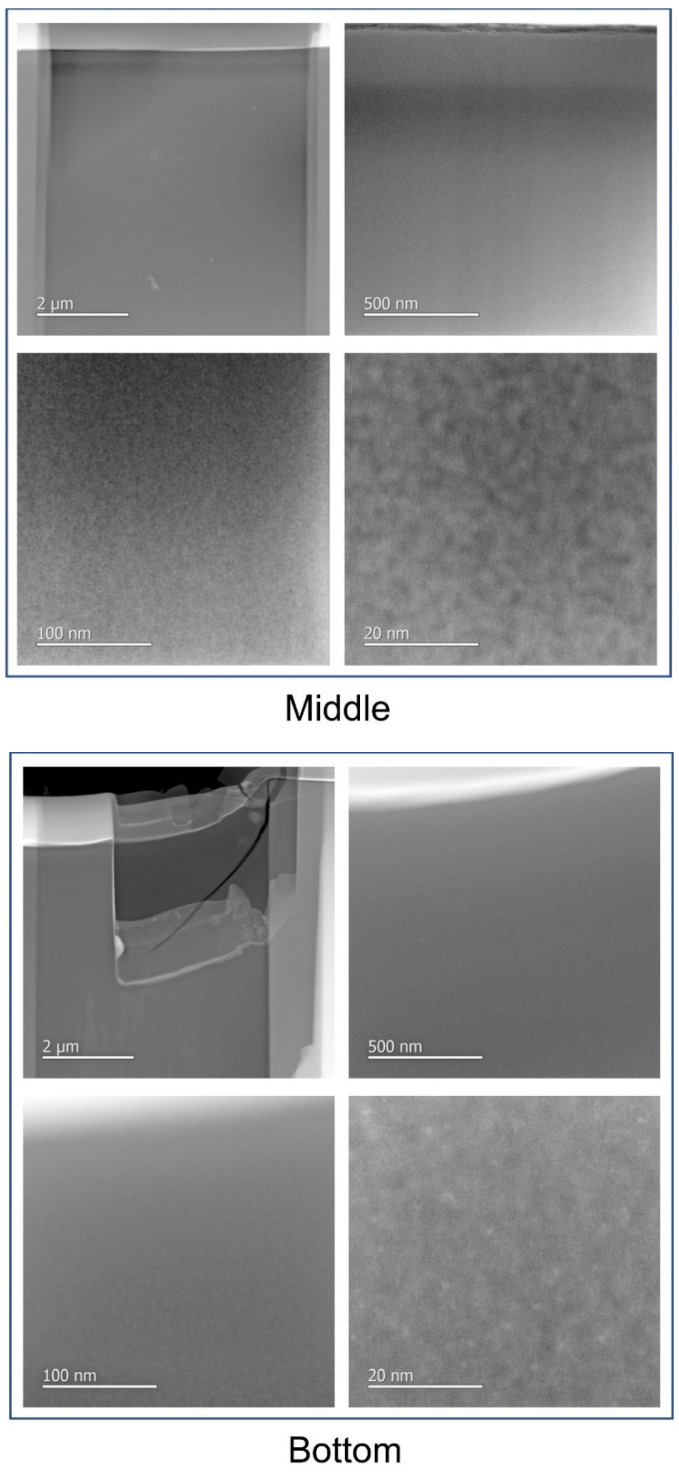
HRTEM images of the samples prepared by FIB.

In order to detect potential aggregates of cluster units in the film, a second approach was implemented by using a high-resolution transmission electron microscope (HRTEM). The analyzed sample is prepared using a focused ion beam (FIB) in order to obtain very thin layers observable with HRTEM. Three cross-sections of film of approximately 10 µm × 1 µm × 0.1 µm were prepared by this technique, as shown in Figure 4. Since it is desired to assess the homogeneity of the film, the three sections are taken from different areas: a first in contact with the substrate (“bottom”), a second in the middle (“middle”) and a last at the interface between the film and the air (“top”) (Figure 4). Once thinned and sized using the FIB, the film sections are deposited on a copper grid to be observed by HRTEM (Figure 5).

Although the brittle nature of the film limited their thinning and it was difficult to obtain perfectly flat surfaces, nevertheless, it was not possible to see the presence of aggregates in the film nor to detect the difference between the three analyzed zones, even at high resolution. These results are in good agreement with the excellent optical transmission and low scattering observed by UV-Vis-NIR spectrometry and could confirm their good dispersion.

Nevertheless, a critical point of this chemical process could be the drying step. Regarding the drying step, the films were subjected to different drying conditions: at ambient temperature for 24 h, or at 50° C for two hours or hundred hours. The Figure 3c and Figure S10 presents the results obtained with the films containing the {Nb_5_TaCl^i^_12_}^n+^ cluster cores (n = 2 and 3) as an example. Unfortunately, as observed on Figure 3c, during the drying process, the cluster units tend to oxidize even at 50 °C. Indeed, we observed a gradual color change, from green to orange, which reflects a clear oxidation of the cluster units and therefore, a modification of the absorption properties. The UV-Vis-NIR spectra in the transmission of the film (See Figure S10) confirm this oxidation of the clusters, from VEC = 16 ({Nb_5_TaCl^i^_12_}^2 +^) to VEC = 15 ({Nb_5_TaCl^i^_12_}^3 +^), in agreement with the UV-Vis-NIR absorption spectra recorded after the dissolution of the K_4_[{Nb_5_TaCl^i^_12_}Cl^a^_6_] cluster compound in acetone [31]. As discussed briefly above, the objective of these studies is to combine the best properties of absorption in the UV and NIR and increase concomitantly the transmission in the visible spectrum. By using this process based on the SiO_2_-PEG matrix, this could be simply achieved by using the partially oxidized (VEC = 15) Ta rich cluster units (x = 6) [22]. Nevertheless, we tried different experimental conditions by exchanging hydrochloric acid with nitric acid, acetic acid or oxalic acid or by using aluminum metal or tin halides as a reducing agent without any real possible improvement after drying, except for aluminum metal in combination with hydrochloric acid [22]. These points on the redox stability and control of the nanoclusters will be developed in the next section in more detail.

### 3.3. Study of the {Nb_6-x_Ta_x_X^i^_12_}-Y@PVP Films

PVP is the second example of matrixes used in these works, and it is often a good candidate complementary to the SiO_2_-PEG matrix. Indeed, the PVP is a nontoxic, biodegradable and biocompatible polymer with an interesting temperature resistance and pH stability, recently used for energy and optical applications [11,12,14,30,32,33,39,40,41,42,43,44,45,46,47]. These results, especially thus on thin films, have shown that it is clearly possible to incorporate inorganic nanoparticles or nanoclusters directly at high concentrations into a PVP matrix without observing any segregation between the organic and inorganic phases. Moreover, PVP is a polymer soluble in several types of solvents, which could be an advantage for the development of solar control materials based on transition metal clusters. First of all, metal atom clusters are soluble in various kinds of solvents depending on the ligands and their optical properties being largely influenced by certain solvents as demonstrated above. It gives the possible control and modulation of their optical properties by choosing the appropriate experimental conditions. Our previous works have shown that the choice to dissolve clusters and PVP in ethanol can facilitate film shaping and accelerate evaporation [11,12,14,30,39,42]. In this new work, we have chosen to favor solubilization of PVP and cluster compounds in water, on the one hand, to prevent the oxidation of the clusters as reported above and, on the other hand, to facilitate the coating as films on glass substrates. The {Nb_6-x_Ta_x_X^i^_12_}-Y@PVP thin films, prepared by drop-casting using PVP water solutions, were first structurally and chemically analyzed to determine their homogeneity and uniformity. Different concentrations from 5 to 50%_wt_ and molar mass (10,000 g∙mol^−1^, 40,000 g∙mol^−1^, 1,300,000 g∙mol^−1^) of PVP were tested. Films with a high mass percentage have better homogeneity, up to a certain limit, because when the solution becomes too viscous, it is complicated to produce a homogeneous deposit of a few tens of micrometers. In order to obtain a suitable viscosity without diluting the cluster units too much, PVP_1M3 was preferred to the other two. Finally, the mass percentage selected for the rest of the work is 10%_wt_, for reasons of viscosity and film thickness. Raman vibrational and optical properties of the films were characterized to evaluate the cluster unit oxidation state and compared to the literature [8,9,30]. The Raman vibrational frequencies of the {Nb_6−x_Ta_x_Cl^i^_12_}-12@PVP films were studied and are very close to the {Nb_6_Cl^i^_12_}-12@PVP (x = 0) and {Ta_6_Cl^i^_12_}-12@PVP (x = 6) films, depending on the value of x (Table S3 and Figure S12) [22,30]. These results confirmed the integrity of the {Nb_6−x_Ta_x_Cl^i^_12_}^2+^ cluster cores.

**Figure 6:**
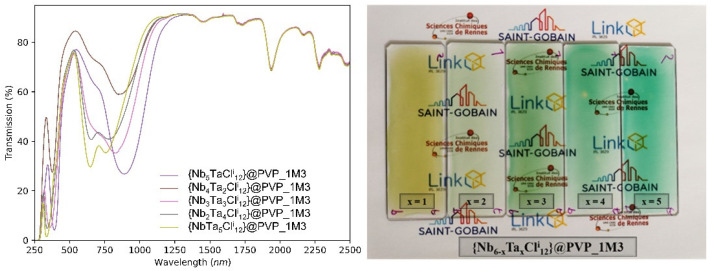
Camera image (**right**) and UV-Vis-NIR transmission spectra (**left**) of the {Nb_6−x_Ta_x_Cl^i^_12_}-5@PVP nanocomposite films deposited on glass substrates (sample sizes are 2.5 cm × 7.5 cm).

Figure S11 shows the cross-sectional optical image of the film {Nb_5_TaBr^i^_12_}-2@PVP and reveals a dense and quite homogeneous film with an average thickness of 44 µm (± 5 µm). The concentration of the nanoclusters has no impact on the thickness in the studied range; only the molar mass, the concentration of PVP (%_wtPVP_) and the deposited volume are the important parameters. The camera image of films (obtained from a water solution containing 5 g∙L^−1^ of K_4_[{Nb_6−x_Ta_x_Cl^i^_12_}Cl^a^_6_]) as well as the UV-Vis spectra in transmission are shown in Figure 6. The spectra agree perfectly with the absorption spectra of the aqueous solutions (Figure 2). We can note that the films are quite homogeneous despite some imperfections related to the evaporation of the solvent. As observed in Figure 6, a large variety of colors, from yellow to green, can be achieved with a very good transparency in the visible range.

**Figure 7:**
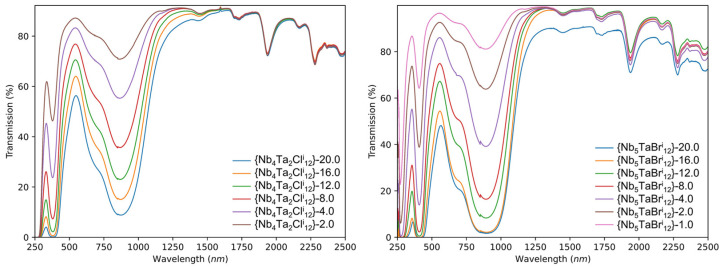
UV-Vis-NIR transmission spectra of the {Nb_6-x_Ta_x_X^i^_12_}-Y@PVP (X = Cl, Br; x = 1, 2) nanocomposite films for concentrations ranging from 1 to 20 g∙L^−1^.

The good transmission in the visible range for these nanocomposite thin films is attributed to good dispersion in the polymer matrix and very low absorption and the size of the nanoclusters (i.e., very low scattering losses as suggested by Rayleigh’s theory). In parallel, good absorption in the UV and NIR is controlled and tuned by the chemical formula of the cluster units. As expected, the higher the cluster concentration of the deposition solution, the higher the absorption intensity in UV and NIR ranges. See, for example, the UV-Vis-NIR transmission spectra of the bromine (x = 1) and chlorine (x = 2) nanocomposite films for concentrations ranging from 1 to 20 g∙L^−1^ (Figure 7 and Figure S13). Moreover, the position of the UV and NIR bands at maximum absorption does not change according to the concentration of the metal atom clusters, which confirms the VEC = 16 and the stability of the nanoclusters in the PVP matrix (Figure 7). We should notice that at a high concentration of the cluster (Y = 20), the stability and the optical properties of the film are impacted after several weeks by a large amount of KCl [30]. The use of the [{Nb_6−x_Ta_x_X^i^_12_}X^a^_2_(H_2_O)^a^_4_]∙4H_2_O aquo-complex compounds as starting materials could easily overlap with this problem.

**Figure 8:**
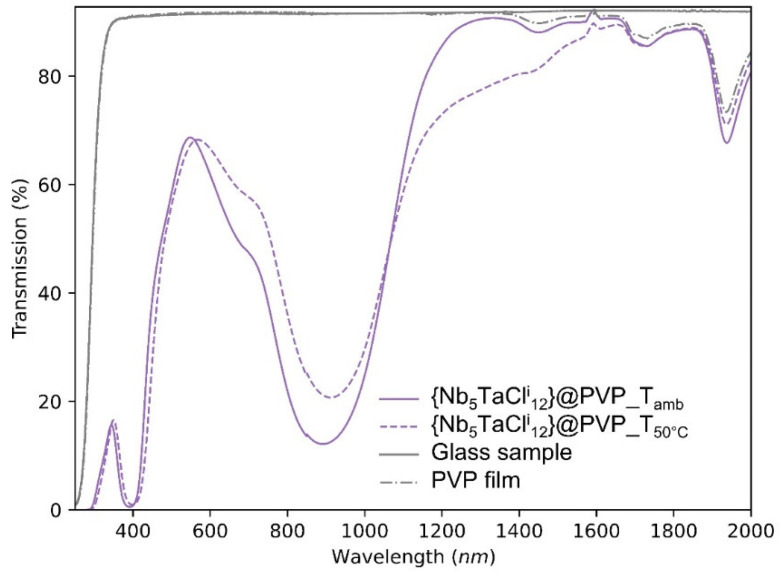
UV-Vis-NIR transmission spectra for the {Nb_5_TaCl^i^_12_}-12@PVP nanocomposite films deposited on glass substrates and drying at room temperature or at 50 °C for 18 h.

Similarly, to the SiO_2_-PEG matrix, the stability of the nanocluster embedded in the PVP matrix was tested. As an example, the {Nb_5_TaCl^i^_12_}-12@PVP films were dried at 50 °C in air for 18 h and compared with the as-deposited film “control” (Figure 8). We observed a partial oxidation of the {Nb_5_TaCl^i^_12_}^2+^ cluster core incorporated into the PVP matrix by the decrease of the absorption band at 900 nm and the appearance of a shoulder at higher wavelengths (1100–1600 nm). Increasing the temperature or the heating treatment duration could lead to the {Nb_5_TaCl^i^_12_}^3+^ cluster core already observed in previous work [31]. In order to avoid this change in the optical properties during the steps of dissolution, deposition or during drying, the degree of oxidation of the cluster units must be controlled by using a reducing or oxidant agent.

**Figure 9:**
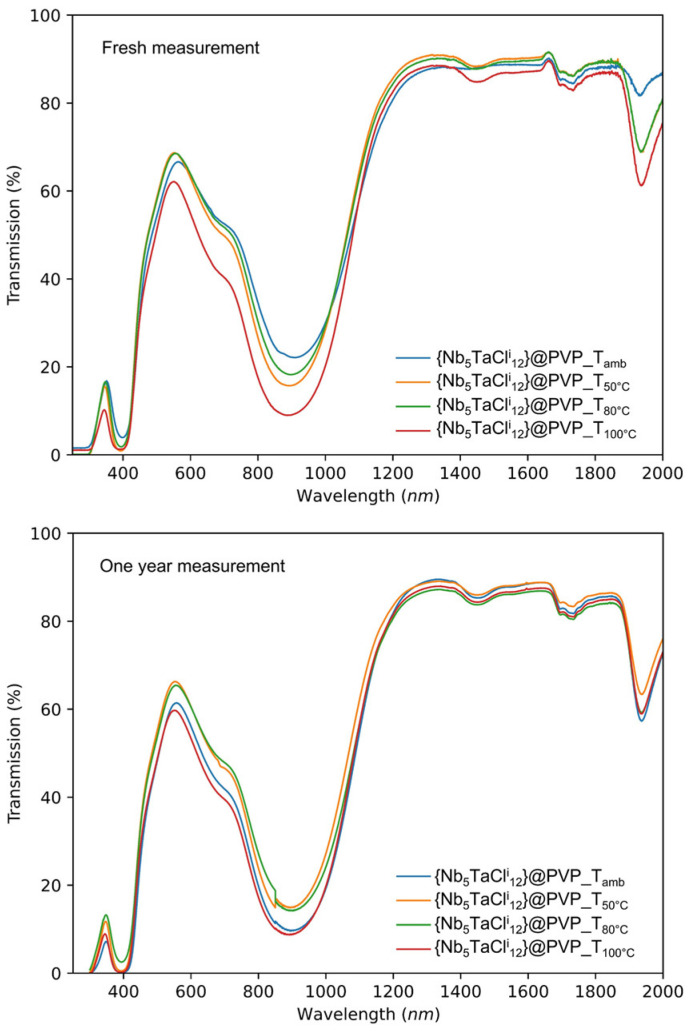
UV-Vis-NIR transmission spectra on glass substrates at room temperature or temperature ranging from 50 °C to 100 °C for the {Nb_5_TaCl^i^_12_}-12.0@PVP nanocomposite films. Left: fresh measurements; right: measurement after one-year aging at room temperature.

In the first case (0 ≤ x ≤ 5), we opt for the use of a reducing agent while in the second case (x = 6), we favor the use of an oxidizing agent. The latter must have an average potential in agreement with the oxidation potentials measured. Indeed, obtaining a {Ta_6_Cl^i^_12_}^4+^ cluster core (VEC = 14), following the dissolution of the K_4_[{Ta_6_Cl^i^_12_}Cl^a^_6_] cluster compound in acetone, for example, is not sought after because the optical properties of these patterns are not in line with the characteristics of a solar control material. In the next section, we will study the various approaches to controlling the reduction or the oxidation of cluster units. The use of a SnBr_2_ (E°(Sn^4 +^/Sn^2 +^) = 0.15 V vs. SCE) as a reducing agent has already been used to maintain the cluster units at VEC = 16, and thus, compensate for the oxidizing conditions of the acid medium at 80 °C, for instance [7,8]. So, in this work, an excess of SnCl_2_∙2H_2_O (m = 250 mg/g of K_4_[{Nb_6−x_Ta_x_X^i^_12_}X^a^_6_]) was added directly with the nanoclusters in the starting aqueous solution. The solution is then filtered, then 10%_wt_ of PVP_1M3 are added and dissolved. After homogenization, the solution is deposited on glass substrates (2.5 cm × 2.5 cm). Once the solvent was evaporated at room temperature and the film has been formed, the substrates are subjected to heat treatments, in order to study the impact of the tin salt on the evolution of the optical properties of the nanoclusters as a function of temperature. The films underwent heat treatments at 50 °C, 80 °C and 100 °C for 18 h, then the optical properties were measured and compared (Figure 9). After one year of storage under laboratory conditions, a new measurement of these spectra is carried out (Figure 9, right). The UV-Vis spectra in transmission show no trace of oxidation of the cluster units. The clusters retain their degree of oxidation and a VEC value of 16, despite the heat treatment, even after one year under ambient conditions of temperature, pressure and humidity. The use of such a reducing agent makes it possible, without any additional step, to avoid any change in the optical properties of the cluster units after heat treatments up to temperatures of, at most, 100 °C. It was also possible to use aluminum metal (E°(Al^3 +^/Al) = −1.662 V vs. SCE) as a reducing agent [48]; but in that specific case, hydrochloric acid must be added in order to “activate” the aluminum metal by removing the oxide layer, which is particularly interesting for the SiO_2_-PEG matrix (see Figure S14).

**Figure 10:**
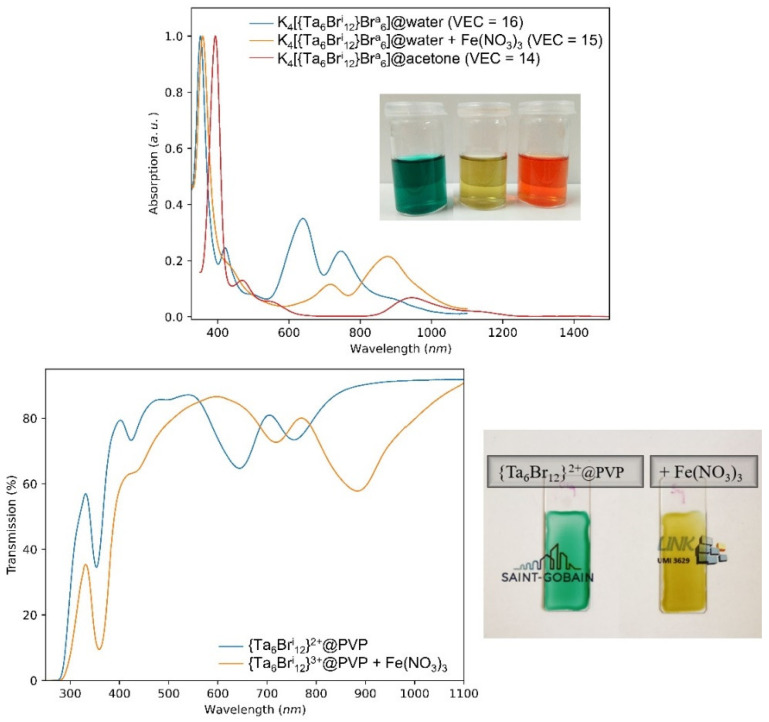
Normalized UV-Vis-NIR absorption spectra of solutions (upper) in water (VEC = 16 and 15) and acetone (VEC = 14) and transmission spectra of the {Ta_6_Br^i^_12_}@PVP nanocomposite films (lower) without (green color, VEC = 16) or with (yellow color, VEC = 15) iron (III) nitrate (**left**, Y = 2; **right**, Y = 10).

The last example concerns the use of iron (III) as oxidizing agent for the tantalum cluster compound [8]. As mentioned previously, we wish to obtain {Ta_6_X^i^_12_}@PVP_1M3 nanocomposite films with a VEC = 15 in order to improve the absorption in the NIR, compared to the cluster units with VEC = 16. The standard potentials of tantalum cluster units in aqueous solution are E°({Ta_6_Br^i^_12_}^3+^/{Ta_6_Br^i^_12_}^2+^) = +0.59 V vs. SHE and E°({Ta_6_Br^i^_12_}^4+^/{Ta_6_Br^i^_12_}^3+^) = +0.89 V vs. SHE [22]. Moreover, we aimed at avoiding VEC = 14 and so we used an oxidizing agent whose potential is lower than that of the couple {Ta_6_Br^i^_12_}^4+^/{Ta_6_Br^i^_12_}^3+^). E°(Fe^3+^/Fe^2+^) = +0.771 V vs. SHE was selected. We should notice that niobium chlorine cluster units have an oxidation potential that is too high (E°({Nb_6_Cl^i^_12_}^3+^/{Nb_6_Cl^i^_12_}^2+^) = +0.83V vs. SHE to be oxidized by iron (III) nitrate. The UV-Vis-NIR absorption spectra of the cluster cores {Ta_6_Br^i^_12_}^n+^ (n = 2, 3 and 4) for different values of VEC (16, 15 and 14, respectively) are shown in Figure 10. As explained previously, the dissolution of K_4_[{Ta_6_Br^i^_12_}Br^a^_6_]. in water leads to patterns of VEC = 16 (green color), then it leads to patterns of VEC = 14 in acetone (red color). As expected, the use of iron (III) nitrate in aqueous solution makes it possible to obtain patterns of VEC = 15 (yellow color). This control of the VEC in solution can be easily transferred to PVP nanocomposite films, as reported in Figure 10. It was clearly demonstrated in this section that by using simple reducing or oxidizing agents, it is possible to control the UV-Vis-NIR optical properties of the nanocomposite films.

In order to evaluate the efficiency of the coating as an energy-saving material, CIE Colorimetric coordinates, different FOM values, T_L_, T_E_, T_L_/T_E_ and S_NIR_ were calculated from the UV-Visible-NIR spectra or haze and clarity were measured [49,50,51,52]. The reflectance of the {Nb_6_Cl_12_}@SiO_2_-PEG coating is reported on Figure S15. The solar transmittance, T_E_, and the NIR shielding ability (S_NIR_) are based on different integrated spectral transmittance of a window weighted with the normalized solar energy distribution spectrum, S. The visible transmittance, T_L_, is calculated in a similar way, but the solar transmittance is now weighted with the photopic response of the human eye, Y (see supplementary information for details). The T_L_ value is an important value and should be higher than 50%, which is the lower limit acceptable for window applications [49,50,51]. For clarity, the results presented in this part will only focus on the measurements carried out on @PVP nanocomposite films which are also easier to shape. The best FOM values (with T_L_ > 50%, except for {Nb_6_Br^i^_12_}-12 (VEC = 16)) are presented in Table 1. All the FOM values for the nanocomposite films with VEC = 16 are available in supporting information in Tables S4 and S5.

**Figure 11:**
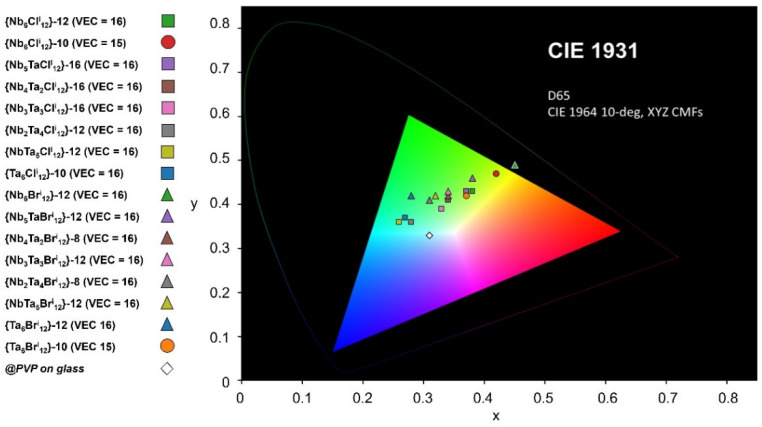
CIE chromaticity coordinates for the nanocomposite films presented in Table 1.

In Table 1, the best T_L_/T_E_ ratios and S_NIR_ (in bold) were obtained for the {Nb_6 × 12_}^2+^ and {Nb_5_TaX_12_}^2+^ cluster cores (X = Cl and Br; x = 1) and decreased with the increase of x for a constant value of VEC = 16. On the contrary, as already discussed, the T_L_/T_E_ value and S_NIR_ strongly increase for the {Ta_6_Br^i^_12_}^3+^ nanocomposite film with VEC = 15 compared to the same cluster core with VEC = 16. The haze and clarity are ranging from 0.56 to 1.14 and 99.2 to 99.5, respectively. Figure 11 and Figure S16 show the CIE chromaticity coordinates of the films reported in Table 1 and for the chlorine series with x = 0, 1, 2 and 6, respectively. These coordinates confirm the large variety of color of the films. For the Nb_6_ chlorine series (x = 0, 1, 2), as expected, the evolution of the color, as a function of the concentration of nanoclusters, is fairly linear, as well as the clusters in the nanocomposites keeping the same VEC (VEC = 16).

## 4. Conclusions

In the frame of the nanoarchitectonic concept, [53] the works carried out concerned the implementation of a new type of material for solar control in order to generate significant energy savings, in particular, in the field of housing. The main objective was to give to the glazing the ability to block solar radiation responsible for the sensation of heat, i.e., radiation whose energy corresponds to the near infrared range (780 nm < NIR < 2500 nm). The new type of materials developed here is the result of the combination between a host matrix (SiO_2_-PEG or PVP), for their easy shaping by solution processes, and K_4_[{Nb_6−x_Ta_x_X^i^_12_}X^a^_6_] (X = Cl, Br, 0 < x ≤ 6) transition metal cluster compounds, for their specific absorption properties. The synthesis protocols for the films have been optimized by considering the sensitivity of the cluster units to oxidation and ligands’ substitution phenomena in solution. The evaluation of the optical performances of the nanocomposites films was carried out through the measurement of FOM values (T_L_, T_E_, T_L_/T_E_ and S_NIR_). The performance depends mainly on the nature of the M/X couple and the oxidation degree of the cluster units. The best performances were obtained for the nanocomposite films {Nb_5_TaCl^i^_12_}^2 +^ @PVP, with a T_L_/T_E_ ratio above 1.30 and a S_NIR_ equal to 54.4%. Such nanocomposite films exhibit a performance equal to or even better than many other solar control materials [54,55,56,57,58,59,60]. It has therefore been demonstrated that the use of cluster compounds as UV and NIR radiation blockers for the development of solar control nanocomposite materials is promising.

## Figures and Tables

**Table 1:** FOM values and CIE color coordinates for the {Nb_6−x_Ta_x_X^i^_12_}-Y@PVP nanocomposite films. The larger the ratio TL/TE, the better. See Ref. [12] for the definition of an “ideal window” TL/TE theoretical value. Best values of this work are highlighted in bold.

Compositions of the Cluster Core in PVP	T_L_	T_E_	T_L_/T_E_	x	y	z	S_NIR_ (%)
**{Nb_6_Cl^i^_12_}-12(VEC = 16) [30]**	**62.2**	**48.3**	**1.29**	0.38	0.43	0.19	**53.7**
{Nb_6_Cl^i^_12_}-10(VEC = 15)	57.1	58.2	0.98	0.42	0.47	0.11	32.0
**{Nb_5_TaCl^i^_12_}-16(VEC = 16) [31]**	**54.1**	**40.7**	**1.33**	0.37	0.43	0.20	**54.4**
{Nb_4_Ta_2_Cl^i^_12_}-16(VEC = 16)	58.1	46.2	1.26	0.34	0.41	0.26	46.2
{Nb_3_Ta_3_Cl^i^_12_}-16(VEC = 16)	61.5	50.8	1.21	0.33	0.39	0.27	40.2
{Nb_2_Ta_4_Cl^i^_12_}-12(VEC = 16)	60.6	57.1	1.06	0.28	0.36	0.36	29.3
{NbTa_5_Cl^i^_12_}-12(VEC = 16)	52.6	52.7	1.00	0.26	0.36	0.38	28.6
{Ta_6_Cl^i^_12_}-10(VEC = 16)	55.6	52.7	1.05	0.27	0.37	0.36	30.7
{Nb_6_Br^i^_12_}-12(VEC = 16)	48.8	37.6	1.29	0.45	0.49	0.10	58.6
**{Nb_5_TaBr^i^_12_}-12(VEC = 16) [31]**	**52.7**	**40.4**	**1.30**	0.38	0.46	0.17	**47.6**
{Nb_4_Ta_2_Br^i^_12_}-8(VEC = 16)	58.6	47.5	1.23	0.34	0.42	0.24	33.1
{Nb_3_Ta_3_Br^i^_12_}-12(VEC = 16)	53.9	45.6	1.18	0.34	0.43	0.24	40.8
{Nb_2_Ta_4_Br^i^_12_}-8(VEC = 16)	55.0	49.8	1.10	0.31	0.41	0.29	33.0
{NbTa_5_Br^i^_12_}-12(VEC = 16)	50.2	45.5	1.10	0.32	0.42	0.26	35.2
{Ta_6_Br^i^_12_}-12(VEC = 16)	55.7	58.1	0.95	0.28	0.42	0.30	18.4
{Ta_6_Br^i^_12_}-10(VEC = 15)	71.2	56.1	1.27	0.37	0.42	0.21	35.8
*@PVP on glass*	*91.4*	*90.5*	*1.01*	*0.31*	*0.33*	*0.35*	*8.3*

## Data Availability

All data concerning this study are contained in the present manuscript, in previous articles or S.I., whose references have been provided.

## Supplementary Materials

The following supporting information can be downloaded at: https://www.mdpi.com/article/10.3390/nano12122052/s1. Figure S1: Mass spectra in positive mode dispersed in water (a) of K_4_[{Nb_6−x_Ta_x_X^i^_12_}X^a^_6_] cluster compounds (X = Cl (spectra 01 to 07)), 0 ≤ x ≤ 6). The [{Nb_5_TaCl^i^_12_}(H_2_O)^a^_5_(OH)^a^]^+^ are indicated by a red circle. Parts of the spectra of (a) are zoomed for clarity (b). (c) Mass spectra in positive mode dispersed in water of K_4_[{Nb_6−x_Ta_x_X^i^_12_}X^a^_6_] cluster compounds (X = Br (spectra 08 to 14), 0 ≤ x ≤ 6). Parts of the spectra of (c) are zoomed for clarity (d). For (b) and (d), the notations above the spectra correspond to the atoms number [Nb; Ta]. The results for x = 0 and 6 are added for clarity. The species are detailed in Table S2; Figure S2: Mass spectra in positive mode dispersed in ethanol (a) of K_4_[{Nb_6−x_Ta_x_X^i^_12_}X^a^_6_] cluster compounds (X = Cl (spectra 01 to 06)), 0 ≤ x ≤ 5). Note that there are no signals for x = 6 in positive mode. Spectra (b) corresponds to the zoom on the 1+ ions area of (a). (c) Mass spectra in positive mode dispersed in ethanol of K_4_[{Nb_6−x_Ta_x_X^i^_12_}X^a^_6_] cluster compounds (X = Br (spectra 08 to 14), 0 ≤ x ≤ 6). Spectra (d) corresponds to the zoom on the 2+ ions area in (c). For (b) and (d), the notations above the spectra correspond to the atoms number [Nb; Ta]. The results for x = 0 and 6 are added for clarity. The species are detailed in Table S2; Figure S3: Mass spectra in negative mode dispersed in acetone (a) of K_4_[{Nb_6−x_Ta_x_X^i^_12_}X^a^_6_] cluster compounds (X = Cl (spectra 01 to 06)), 0 ≤ x ≤ 6. The [TaBr_6_]^1-^are indicated by the red circle A and [Nb_6_Br _15_]^1-^, [Nb_5_Br_15_]^1-^, [NbTa_5_Br_15_]^1-^ by the red circle B. Parts of the spectra of (a) are zoomed for clarity (b). (c) Mass spectra in negative mode dispersed in acetone of K_4_[{Nb_6−x_Ta_x_X^i^_12_}X^a^_6_] cluster compounds (X = Br (spectra 08 to 14), 0 ≤ x ≤ 6). Spectra (d) correspond to the zoom on the 2- ions area of (c). For (b) and (d), the notations above the spectra correspond to the atoms number [Nb; Ta]. The results for x = 0 and 6 are added for clarity. The species are detailed in Table S2; Figure S4: Camera image of SiO_2_-PEG bulk matrix and thin film. UV-Vis-NIR transmission spectra of the @SiO_2_-PEG thin film; Figure S5: Camera image of the homemade upgraded Mayer bar coater (left) and {Nb_6_Cl^i^_12_}@SiO_2_-PEG thin film deposited on 10 cm × 15 cm glass substrate by Mayer bar coater (right). Teflon sample holder (A) (30 cm × 25 cm × 2 cm), glass substrate (B) (max: 15 cm × 10 cm × 0.2 cm) et bare-coater (C); Figure S6: Camera images of the {Nb_6−x_TaBr^i^_12_}@SiO_2_-PEG nanocomposites thin films; Figure S7: UV-Visible-NIR transmission spectra of the films based on the {Nb_6−x_Ta_x_Cl^i^_12_}^2+^ cluster cores (0 ≤ x ≤ 2; VEC = 16) with different thickness; Figure S8: Sketch and digital microscope image of the cross section of a {Nb_6_Cl_12_}@SiO_2_-PEG nanocomposite film deposited on glass substrate; Figure S9: SEM image and EDX analysis of the {Nb_6_Cl_12_}@SiO_2_-PEG nanocomposite film deposited on glass substrate; Figure S10: UV-Vis-NIR transmission spectra of the {Nb_5_TaCl_12_}@SiO_2_-PEG nanocomposite films before and after annealed at 50°C/100h; Figure S11: Example of digital microscope pictures of the cross section of a {Nb_5_TaBr_12_}-2@PVP nanocomposite films on glass substrate; Figure S12: Normalized Raman spectra of the {Nb_6−x_Ta_x_Cl^i^_12_}-12@PVP nanocomposite films (1 ≤ x ≤ 5]. Ref = @PVP film without metal atom clusters; Figure S13: UV-Vis-NIR transmission spectra of the {Nb_6−x_Ta_x_X^i^_12_}-Y@PVP (X = Cl, Br; 0 ≤ x ≤ 6) nanocomposite films for concentrations ranging from 1 to 20 g∙L^−1^; Figure S14: UV-Vis-NIR transmission spectra of the {Nb_5_TaCl_12_}@SiO_2_-PEG nanocomposite films without and with aluminum metal as reducing agent; Figure S15: UV-Vis-NIR transmission and reflectance spectra of the glass substrate and the {Nb_6_Cl_12_}@SiO_2_-PEG nanocomposite films; Figure S16:. CIE chromaticity coordinates for the nanocomposite films based on chlorine cluster compounds with x = 0, 1, 2 and 6; Table S1: Characteristic times for the hydrolysis-condensation process of the SiO_2_-PEG matrix; Table S2: Mass spectra results with major detected ions; Table S3: Main Raman signature of the {Nb_6−x_Ta_x_Cl^i^_12_}-12@PVP nanocomposite films (0 ≤ x ≤ 6. The {Nb_6_Cl^i^_12_}@PVP Raman signature (x = 0) was reported very recently in Ref. [1]; Table S4: FOM for all the SiO_2_-PEG films with VEC = 16; Table S5: FOM for all the PVP films with VEC = 16. References [61,62,63,64,65] are cited in the Supplementary Materials.
