# Supplementary material for: Controlling the Deposition Process of Nanoarchitectonic Nanocomposites Based on {Nb6−xTaxXi12}n+ Octahedral Cluster-Based Building Blocks (Xi = Cl, Br; 0 ≤ x ≤ 6, n = 2, 3, 4) for UV-NIR Blockers Coating Applications
Source: Nanomaterials (Basel). 2022 Jun 15;12(12):2052. doi: 10.3390/nano12122052 (PMC9227475; doi:10.3390/nano12122052)
Supplement: Supplementary file 1 [file nanomaterials-12-02052-s001.zip › nanomaterials-1733950-supplementary.pdf]

nanomaterials

MDPI

Supplementary materials

Controlling the Deposition Process of Nanoarchitectonic Nanocomposites Based on $\{Nb_{6-x}Ta_xX^i_{12}\}^{n+}$ Octahedral Cluster-Based Building Blocks (X^i = Cl, Br; $0 \le x \le 6$, n = 2, 3, 4) for UV-NIR Blockers Coating Applications

Clément Lebastard [1,2,*], Maxence Wilmet [1,2,3], Stéphane Cordier [1], Clothilde Comby-Zerbino [4], Luke MacAleese [4], Philippe Dugourd [4], Toru Hara [5], Naoki Ohashi [2,6], Tetsuo Uchikoshi [2,6] and Fabien Grasset [1,2,*]

1 Univ Rennes, CNRS, ISCR, UMR6226, F-35000 Rennes, France; maxence.wilmet@saint-gobain.com (M.W.); stephane.cordier@univ-rennes1.fr (S.C.)
2 CNRS-Saint Gobain-NIMS, IRL3629, Laboratory for Innovative Key Materials and Structures (LINK), National Institute for Materials Science (NIMS), 1-1 Namiki, Tsukuba 305-0044, Japan; ohashi.naoki@nims.go.jp (N.O.); uchikoshi.tetsuo@nims.go.jp (T.U.)
3 Saint Gobain Research Paris, F-93300 Aubervilliers, France;
4 Univ Lyon, Univ Claude Bernard Lyon 1, CNRS, Institut Lumière Matière, F-69622 Lyon, France; clothilde.comby-zerbino@univ-lyon1.fr (C.C.-Z.); luke.mac-aleese@univ-lyon1.fr (L.M.); philippe.dugourd@univ-lyon1.fr (P.D.)
5 Research Center for Structural Materials, NIMS, 1-2-1 Sengen, Tsukuba, Ibaraki 305-0047, Japan; hara.toru@nims.go.jp (T.H.)
6 Research Center for Functional Materials, NIMS, 1-1 Namiki, Tsukuba, Ibaraki 305-0044, Japan
* Correspondence: lebastard.clementhugo@nims.go.jp (C.L.); fabien.grasset@univ-rennes1.fr (F.G.)

Table S1. Characteristic times for the hydrolysis-condensation process of the SiO_2-PEG matrix.

pH	Before hydrolysis	Before condensation
2	18 h	months
2.5	18 h	several weeks
3	22 h	several days
3.5	∞	2 days
4	∞	42 h
5	∞	42 h
6	∞	42 h
7	∞	42 h

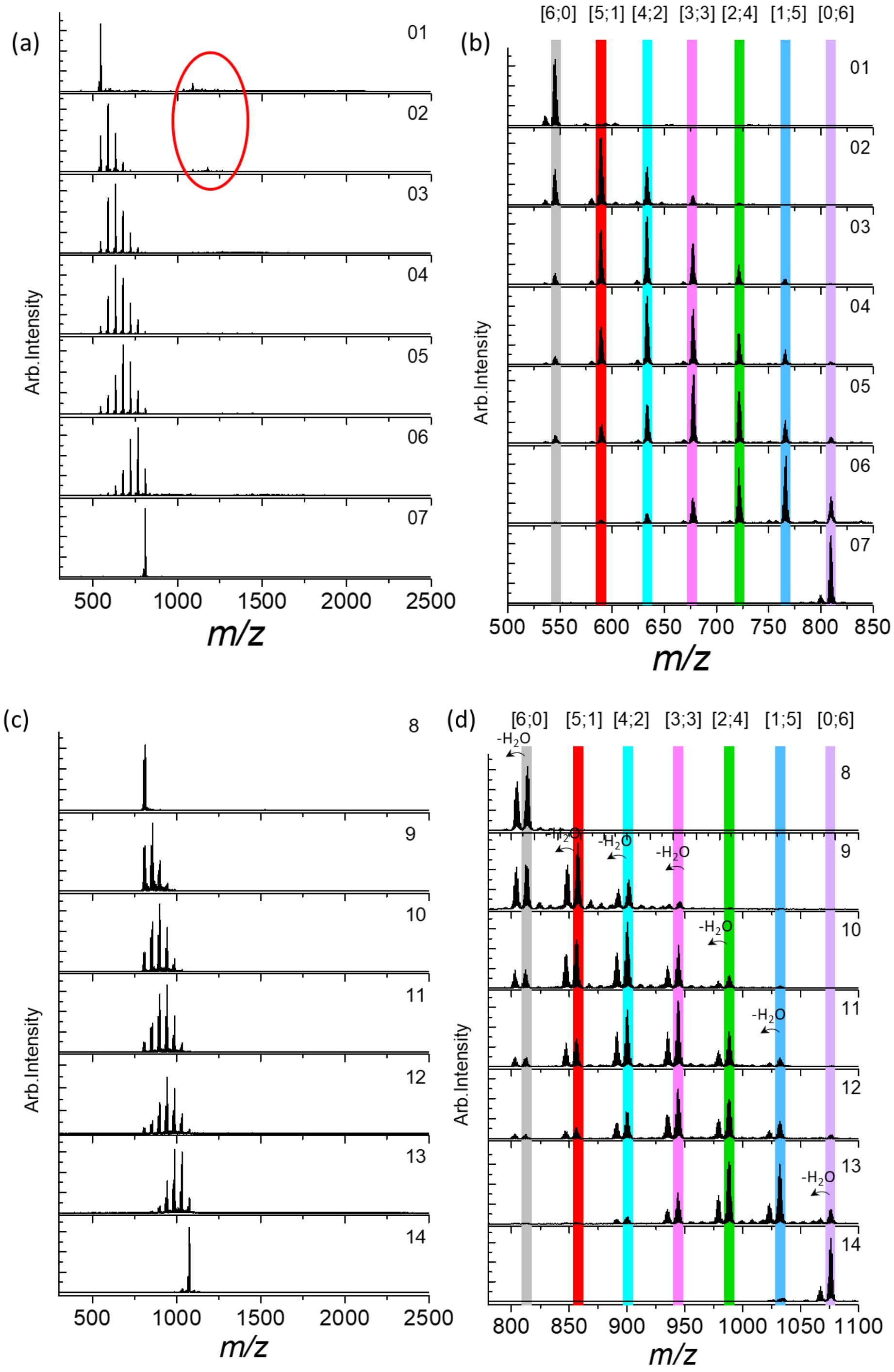

Figure S1. Mass spectra in positive mode dispersed in water (**a**) of $K_4[\{Nb_{6-x}Ta_xCl^i_{12}\}Cl^a_6]$ cluster compounds (spectra 01 to 07)), $0 \leq x \leq 6$). The $[\{Nb_5TaCl^i_{12}\}(H_2O)^a_5(OH)^a]^+$ are indicated by a red circle. Parts of the spectra of (**a**) are zoomed for clarity (**b**). (**c**) Mass spectra in positive mode dispersed in water of $K_4[\{Nb_{6-x}Ta_xBr^i_{12}\}Br^a_6]$ cluster compounds (spectra 08 to 14), $0 \leq x \leq 6$). Parts of the spectra of (**c**) are zoomed for clarity (**d**). For (**b**) and (**d**), the notations above the spectra correspond to the atoms number [Nb; Ta]. The results for x = 0 and 6 are added for clarity. The species are detailed in table S2.

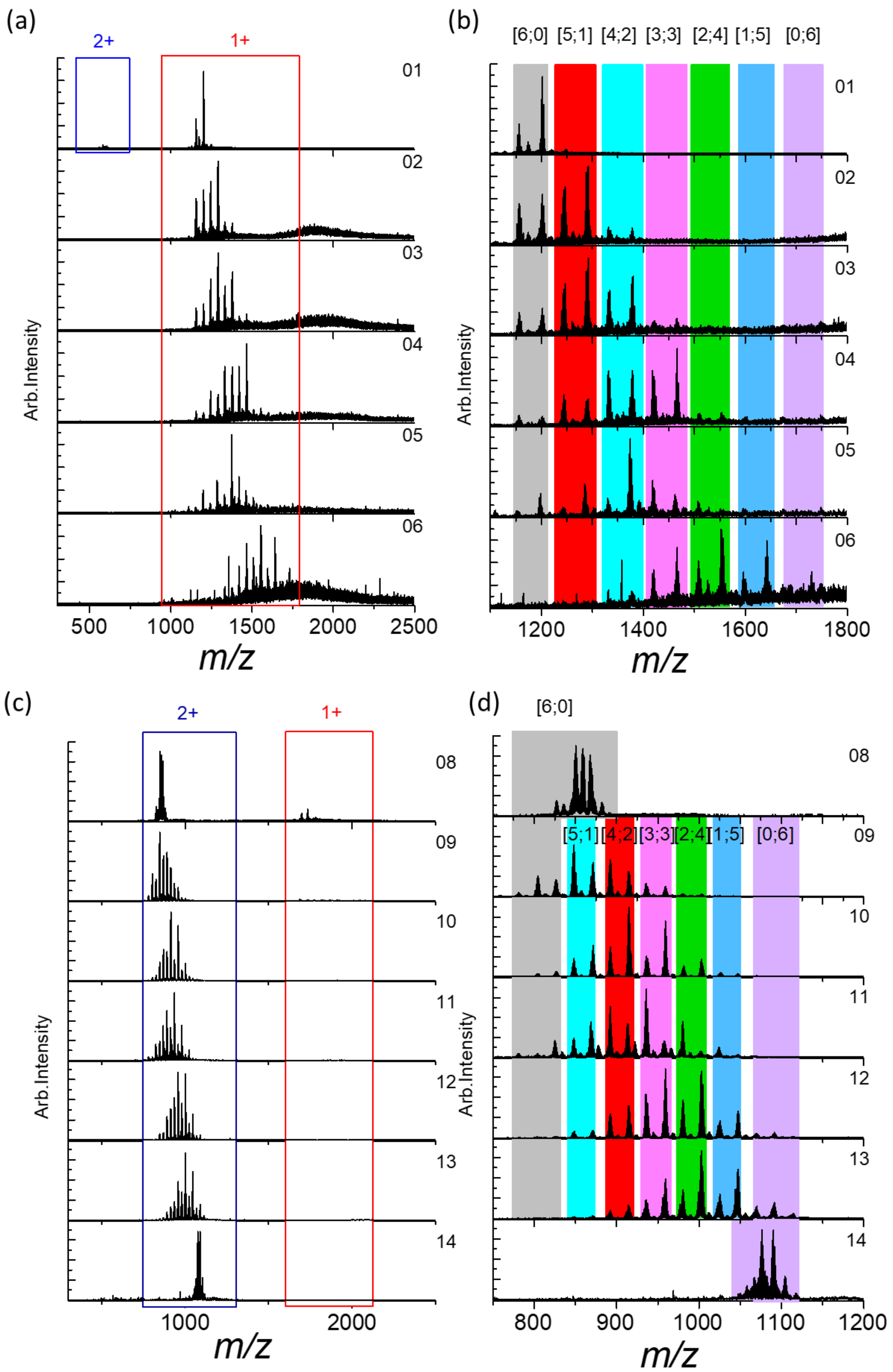

Figure S2. Mass spectra in positive mode dispersed in ethanol (**a**) of $K_4[\{Nb_{6-x}Ta_xCl^i_{12}\}Cl^a_6]$ cluster compounds (spectra 01 to 06)), $0 \leq x \leq 5$). Note that there are no signals for x=6 in positive mode. Spectra (**b**) corresponds to the zoom on the 1+ ions area of (**a**). (**c**) Mass spectra in positive mode dispersed in ethanol of $K_4[\{Nb_{6-x}Ta_xBr^i_{12}\}Br^a_6]$ cluster compounds (spectra 08 to 14), $0 \leq x \leq 6$). Spectra (**d**) corresponds to the zoom on the 2+ ions area in (**c**). For (**b**) and (**d**), the notations above the spectra correspond to the atoms number [Nb; Ta]. The results for x = 0 and 6 are added for clarity. The species are detailed in table S2.

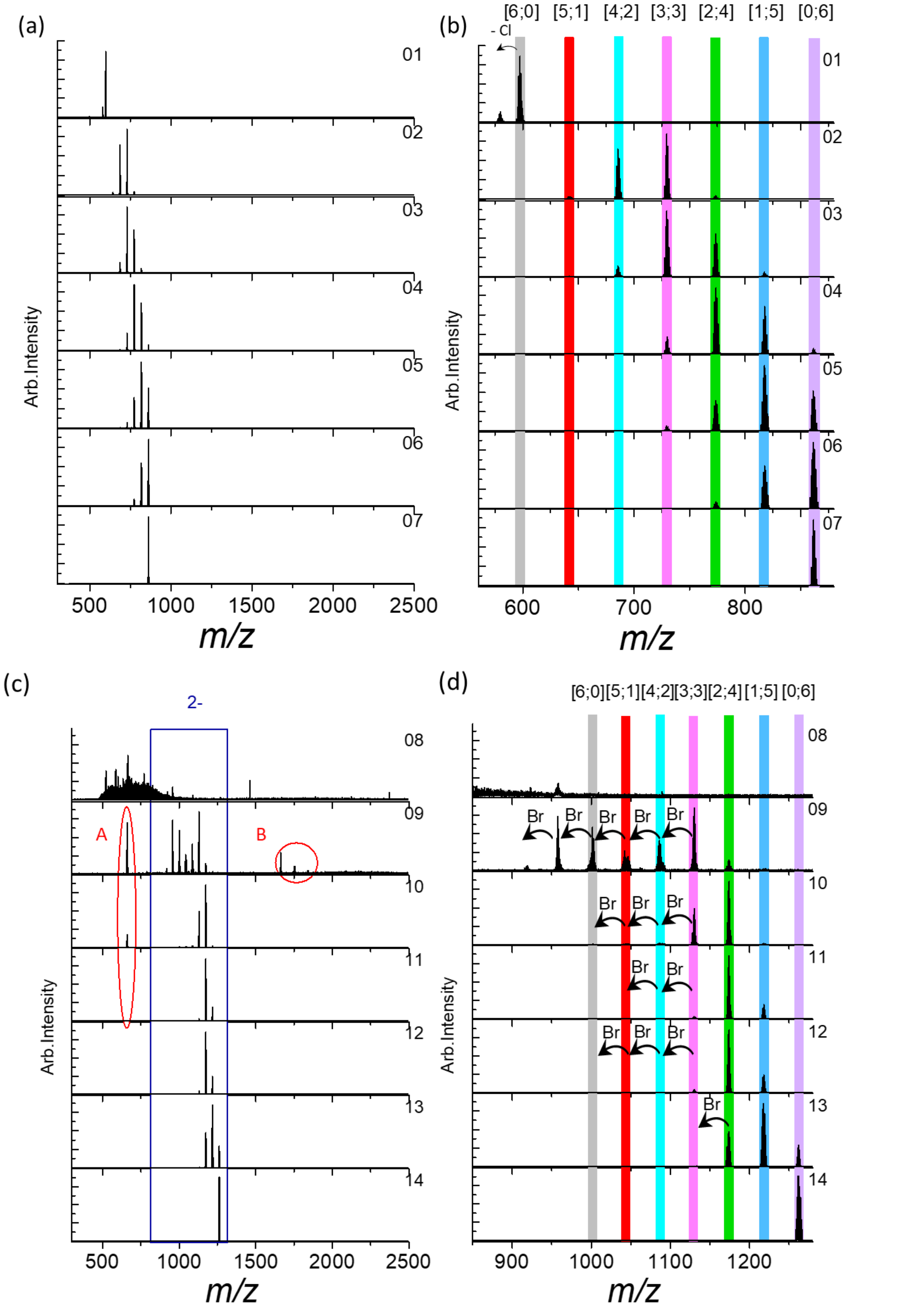

Figure S3. Mass spectra in negative mode dispersed in acetone (**a**) of $K_4[\{Nb_{6-x}Ta_xCl^i_{12}\}Cl^a_6]$ cluster compounds (spectra 01 to 06)), $0 \leq x \leq 6$. The $[TaBr_6]^{1-}$are indicated by the red circle A and $[Nb_6Br_{15}]^{1-}$, $[Nb_5Br_{15}]^{1-}$, $[NbTa_5Br_{15}]^{1-}$ by the red circle B. Parts of the spectra of (**a**) are zoomed for clarity (**b**). (**c**) Mass spectra in negative mode dispersed in acetone of $K_4[\{Nb_{6-x}Ta_xBr^i_{12}\}Br^a_6]$ cluster compounds (spectra 08 to 14), $0 \leq x \leq 6$). Spectra (**d**) correspond to the zoom on the 2- ions area of (**c**). For (**b**) and (**d**), the notations above the spectra correspond to the atoms number [Nb; Ta]. The results for x = 0 and 6 are added for clarity. The species are detailed in table S2.

Table S2. Mass spectra results with major detected ions.

Cluster compounds	Solvent	Positive mode	Negative mode
$K_4Nb_5TaCl_{18}$	Water	$[\{Nb_5TaCl^i_{12}\}(H_2O)^a_6]^{2+}$ (major)<br>$[\{Nb_5TaCl^i_{12}\}(H_2O)^a_5(OH)^a]^+$ (minor)<br>$[\{Nb_6Cl^i_{12}\}(H_2O)^a_6]^{2+}$ (minor)<br>$[\{Nb_4Ta_2Cl^i_{12}\}(H_2O)^a_6]^{2+}$ (minor)	-
	Ethanol	$[Nb_5TaCl_{12}(H_2O)_n(OH)(EtOH)_m]^{1+}$ (major)<br>$[Nb_6Cl_{12}(H_2O)_n(OH)(EtOH)_m]^{1+}$ (minor)<br>n = 1, 2; m = 3, 4 and n+m = 5	-
	Acetone	-	$[\{Nb_4Ta_2Cl^i_{12}\}Cl^a_6]^{2-}$ (major)<br>$[\{Nb_3Ta_3Cl^i_{12}\}Cl^a_6]^{2-}$ (major)
$K_4Nb_4Ta_2Cl_{18}$	Water	$[\{Nb_4Ta_2Cl^i_{12}\}(H_2O)^a_6]^{2+}$ (major)<br>$[\{Nb_5TaCl^i_{12}\}(H_2O)^a_6]^{2+}$ (minor)	-
	Ethanol	$[Nb_5TaCl_{12}(H_2O)_n(OH)(EtOH)_m]^{1+}$ (major)<br>$Nb_4Ta_2Cl_{12}(H_2O)_n(OH)(EtOH)_m]^{1+}$ (minor)<br>n = 1, 2; m = 3, 4 and n+m = 5	-
	Acetone	-	$[\{Nb_3Ta_3Cl^i_{12}\}Cl^a_6]^{2-}$ (major)<br>$[\{Nb_2Ta_4Cl^i_{12}\}Cl^a_6]^{2-}$ (minor)
$K_4Nb_3Ta_3Cl_{18}$	Water	$[\{Nb_3Ta_3Cl^i_{12}\}(H_2O)^a_6]^{2+}$ (minor)<br>$[\{Nb_2Ta_4Cl^i_{12}\}(H_2O)^a_6]^{2+}$ (major)	-
	Ethanol	$[Nb_3Ta_3Cl_{12}(H_2O)_n(OH)(EtOH)_m]^{1+}$ (major)<br>$[Nb_4Ta_2Cl_{12}(H_2O)_n(OH)(EtOH)_m]^{1+}$ (minor)<br>n = 1, 2; m = 3, 4 and n+m = 5	-
	Acetone	-	$[\{Nb_3Ta_3Cl^i_{12}\}Cl^a_6]^{2-}$ (minor)<br>$[\{Nb_2Ta_4Cl^i_{12}\}Cl^a_6]^{2-}$ (major)<br>$[\{Nb_1Ta_5Cl^i_{12}\}Cl^a_6]^{2-}$ (minor)
$K_4Nb_2Ta_4Cl_{18}$	Water	$[\{Nb_2Ta_4Cl^i_{12}\}(H_2O)^a_6]^{2+}$ (minor)<br>$[\{NbTa_5Cl^i_{12}\}(H_2O)^a_6]^{2+}$ (major)	-
	Ethanol	$[Nb_4Ta_2Cl_{12}(H_2O)_n(OH)(EtOH)_m]^{1+}$ (major)<br>$[Nb_3Ta_3Cl_{12}(H_2O)_n(OH)(EtOH)_m]^{1+}$ (major)<br>n = 1, 2; m = 3, 4 and n+m = 5	-
	Acetone	-	$[\{Nb_2Ta_4Cl^i_{12}\}Cl^a_6]^{2-}$ (minor)<br>$[\{Nb_1Ta_5Cl^i_{12}\}Cl^a_6]^{2-}$ (major)<br>$[\{Ta_6Cl^i_{12}\}Cl^a_6]^{2-}$ (minor)
$K_4NbTa_5Cl_{18}$	Water	$[\{NbTa_5Cl^i_{12}\}(H_2O)^a_6]^{2+}$(major)<br>$[\{Nb_2Ta_4Cl^i_{12}\}(H_2O)^a_6]^{2+}$ (minor)	-
	Ethanol	$[Nb_3Ta_3Cl_{12}(H_2O)_n(OH)(EtOH)_m]^{1+}$ (minor)<br>$Nb_2Ta_4Cl_{12}(H_2O)_n(OH)(EtOH)_m]^{1+}$ (major)<br>$[NbTa_5Cl_{12}(H_2O)_n(OH)(EtOH)_m]^{1+}$	-

		n = 1, 2; m = 3, 4 and n+m = 5	
	Acetone	-	$[\{NbTa_5Cl^{i}_{12}\}Cl^{a}_{6}]^{2-}$ (minor)<br>$[\{Ta_6Cl^{i}_{12}\}Cl^{a}_{6}]^{2-}$ (major)
$K_4Nb_5TaBr_{18}$	Water	$[\{Nb_5TaBr^{i}_{12}\}(H_2O)^{a}_{6}]^{2+}$ (major)<br>$[\{Nb_6Br^{i}_{12}\}(H_2O)^{a}_{6}]^{2+}$ (minor)	-
	Ethanol	$[Nb_5TaBr_{12}(H_2O)_n(EtOH)_m]^{2+}$ (major)<br>$[Nb_2Ta_4Br_{12}(H_2O)_n(EtOH)_m]^{2+}$ (minor)<br>n = 5, 6; m = 0, 1 and n+m = 6	-
	Acetone	-	$[\{Nb_6Br^{i}_{12}\}Br^{a}_{6}]^{2-}$ (minor)<br>$[\{Nb_5Ta_2Br^{i}_{12}\}Br^{a}_{6}]^{2-}$ (minor)<br>$[\{Nb_4Ta_2Br^{i}_{12}\}Br^{a}_{6}]^{2-}$ (minor)<br>$[\{Nb_3Ta_3Br^{i}_{12}\}Br^{a}_{6}]^{2-}$ (major)<br>$[NbBr_6]^{1-}$ (A circle in red)<br>$[Nb_6Br_{15}]^{1-}$, $[Nb_5Br_{15}]^{1-}$, $[Nb_5TaBr_{15}]^{1-}$ (B circle in red)
$K_4Nb_4Ta_2Br_{18}$	Water	$[\{Nb_4Ta_2Br^{i}_{12}\}(H_2O)^{a}_{6}]^{2+}$(major)<br>$[\{Nb_5TaBr^{i}_{12}\}(H_2O)^{a}_{6}]^{2+}$ (minor)	-
	Ethanol	$[Nb_2Ta_4Br_{12}(H_2O)_n(EtOH)_m]^{2+}$ (major)<br>$[Nb_3Ta_3Br_{12}(H_2O)_n(EtOH)_m]^{2+}$ (minor)<br>n = 5, 6; m = 0, 1 and n+m = 6	-
	Acetone	-	$[\{Nb_3Ta_3Br^{i}_{12}\}Br^{a}_{6}]^{2-}$ (minor)<br>$[\{Nb_2Ta_4Br^{i}_{12}\}Br^{a}_{6}]^{2-}$ (major)<br>$[NbBr_6]^{1-}$ (A circle in red)
$K_4Nb_3Ta_3Br_{18}$	Water	$[\{Nb_3Ta_3Br^{i}_{12}\}(H_2O)^{a}_{6}]^{2+}$(major)<br>$[\{Nb_4Ta_2Br^{i}_{12}\}(H_2O)^{a}_{6}]^{2+}$ (minor)	-
	Ethanol	$[Nb_2Ta_4Br_{12}(H_2O)_n(EtOH)_m]^{2+}$ (minor)<br>$[Nb_3Ta_3Br_{12}(H_2O)_n(EtOH)_m]^{2+}$ (major)<br>n = 5, 6; m = 0, 1 and n+m = 6	-
	Acetone	-	$[\{Nb_3Ta_3Br^{i}_{12}\}Br^{a}_{6}]^{2-}$ (minor)<br>$[\{Nb_2Ta_4Br^{i}_{12}\}Br^{a}_{6}]^{2-}$ (major)<br>$[\{NbTa_5Br^{i}_{12}\}Br^{a}_{6}]^{2-}$ (minor)
$K_4Nb_2Ta_4Br_{18}$	Water	$[\{Nb_2Ta_4Br^{i}_{12}\}(H_2O)^{a}_{6}]^{2+}$(major)<br>$[\{Nb_3Ta_3Br^{i}_{12}\}(H_2O)^{a}_{6}]^{2+}$ (major)<br>$[\{Nb_4Ta_2Br^{i}_{12}\}(H_2O)^{a}_{6}]^{2+}$ (minor)	-
	Ethanol	$[Nb_3Ta_3Br_{12}(H_2O)_n(EtOH)_m]^{2+}$ (major)<br>$[Nb_2Ta_4Br_{12}(H_2O)_n(EtOH)_m]^{2+}$ (major)<br>n = 5, 6; m = 0, 1 and n+m = 6	-
	Acetone	-	$[\{Nb_2Ta_4Br^{i}_{12}\}Br^{a}_{6}]^{2-}$ (major)<br>$[\{NbTa_5Br^{i}_{12}\}Br^{a}_{6}]^{2-}$ (minor)
$K_4NbTa_5Br_{18}$	Water	$[\{NbTa_5Br^{i}_{12}\}(H_2O)^{a}_{6}]^{2+}$(major)<br>$[\{Nb_2Ta_4Br^{i}_{12}\}(H_2O)^{a}_{6}]^{2+}$ (major)	-

		$[\{Nb_3Ta_3Br^i_{12}\}(H_2O)^a_6]^{2+}$ (minor)	
	Ethanol	$[Nb_2Ta_4Br_{12}(H_2O)_n(EtOH)_m]^{2+}$ (major)<br>$[NbTa_5Br_{12}(H_2O)_n(EtOH)_m]^{2+}$ (minor)<br>n = 5, 6; m = 0, 1 and n+m = 6	-
	Acetone	-	$[\{NbTa_5Br^i_{12}\}Br^a_6]^{2-}$ (major)<br>$[\{Ta_6Br^i_{12}\}Br^a_6]^{2-}$ (minor)<br>$[\{Nb_2Ta_4Br^i_{12}\}Br^a_6]^{2-}$ (minor)

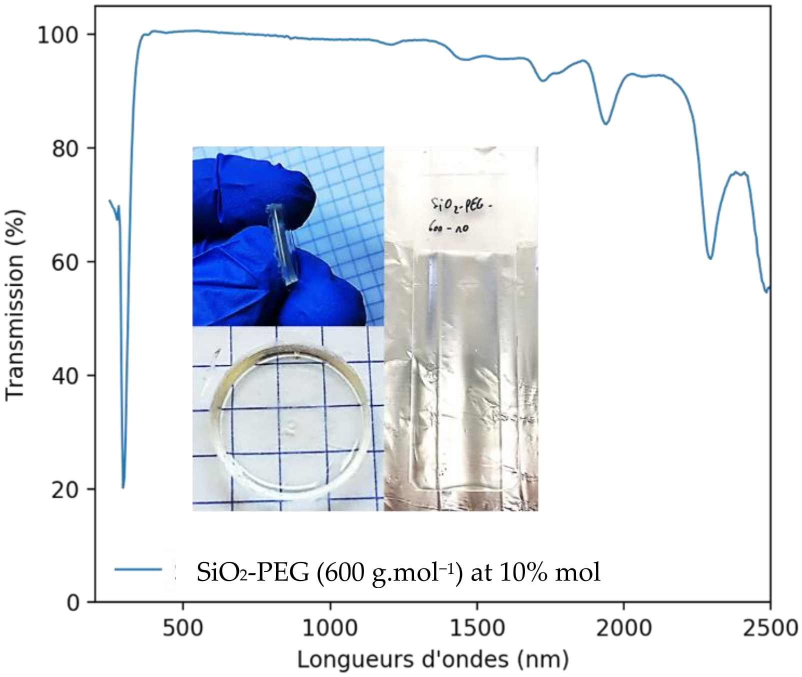

Figure S4. Camera image of SiO_2-PEG bulk matrix and thin film. UV-Vis-NIR transmission spectra of the @SiO_2-PEG thin film.

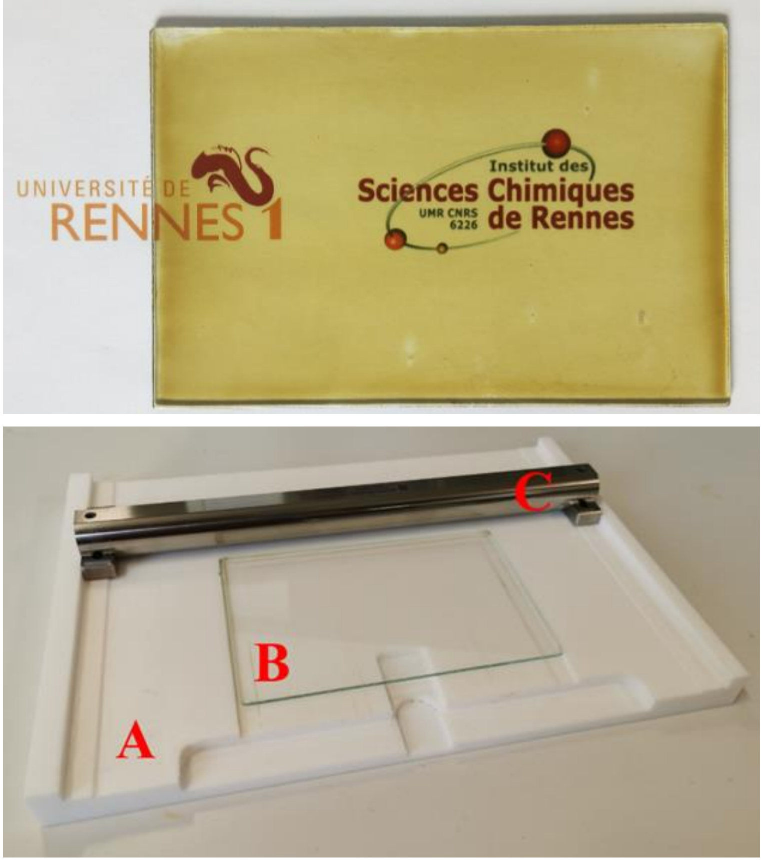

Figure S5. Camera image of the homemade upgraded Mayer bar coater (left) and $\{Nb_6Cl^i_{12}\}$@SiO_2-PEG thin film deposited on 10 cm × 15 cm glass substrate by Mayer bar coater (right). Teflon sample holder (A) (30 cm × 25 cm × 2 cm), glass substrate (B) (max: 15 cm × 10 cm × 0.2 cm) et bare-coater (C).

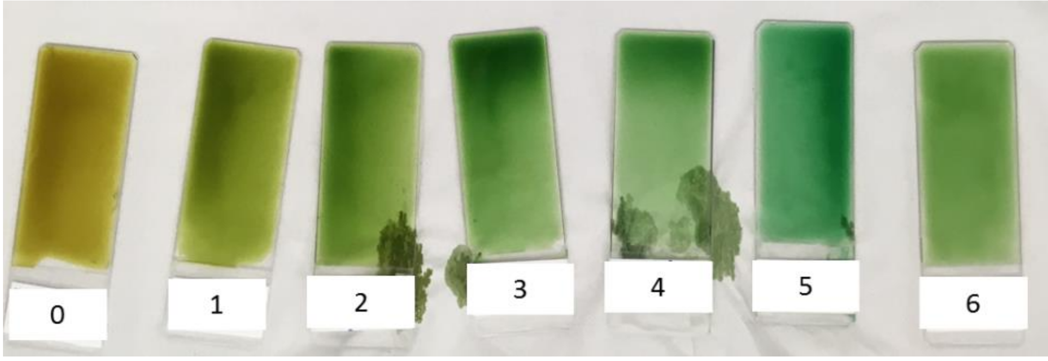

Figure S6. Camera images of the $\{Nb_{6-x}Ta_xBr^i_{12}\}$@$SiO_2$-PEG nanocomposites thin films.

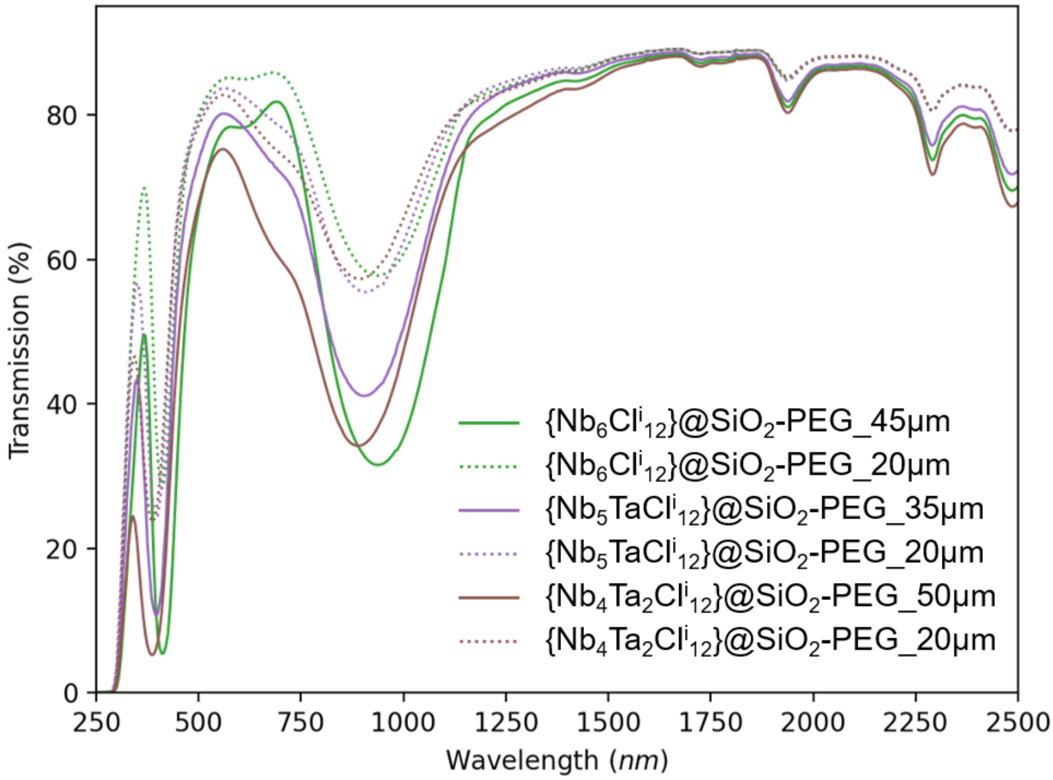

Figure S7. UV-Visible-NIR transmission spectra of the films based on the $\{Nb_{6-x}Ta_xCl^i_{12}\}^{2+}$ cluster cores ($0 \leq x \leq 2$; VEC = 16) with different thickness.

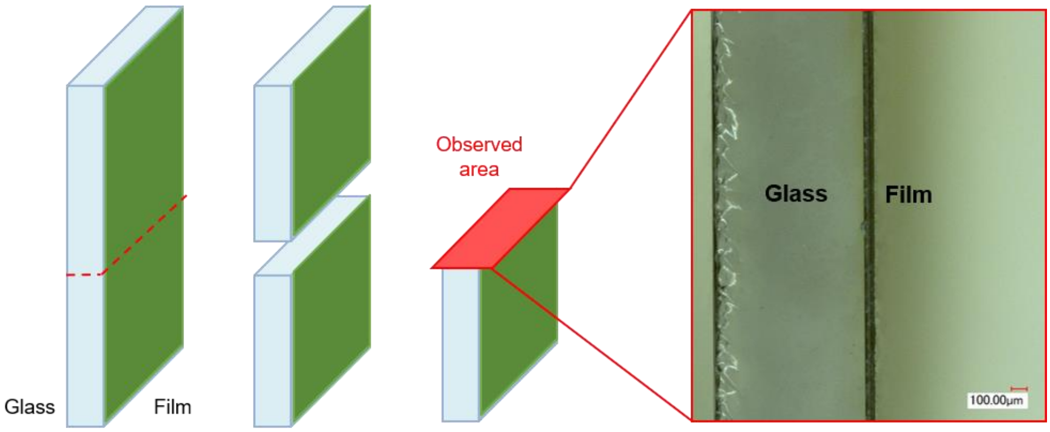

Figure S8. Sketch and digital microscope image of the cross section of a $\{Nb_6Cl_{12}\}$@SiO_2-PEG nanocomposite film deposited on glass substrate.

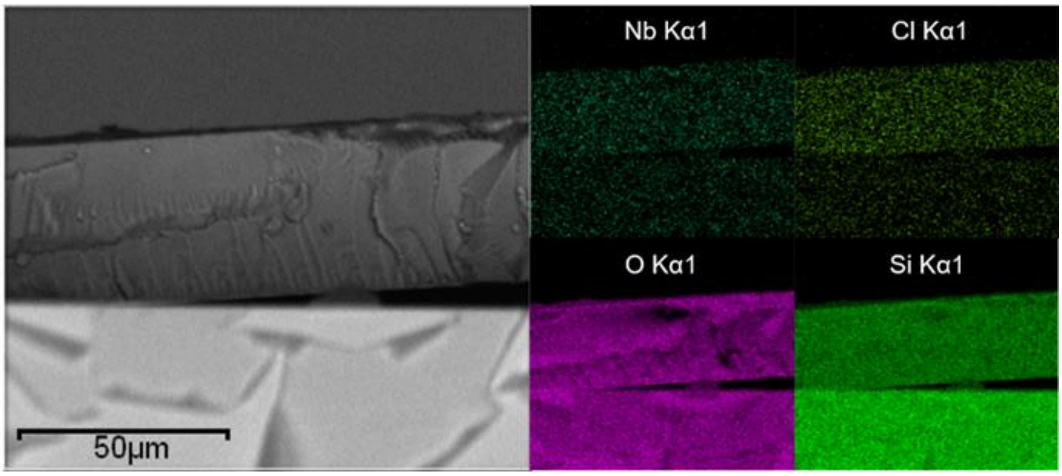

Figure S9. SEM image and EDX analysis of the $\{Nb_6Cl_{12}\}@SiO_2$-PEG nanocomposite film deposited on glass substrate.

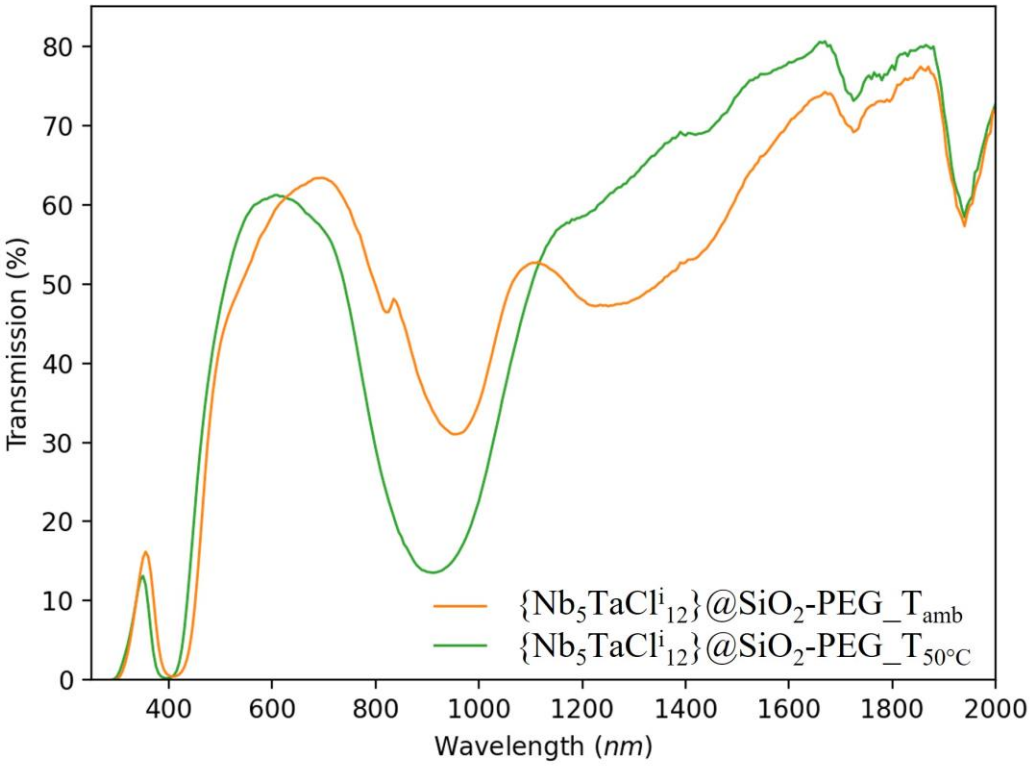

Figure S10. UV-Vis-NIR transmission spectra of the $\{Nb_5TaCl_{12}\}@SiO_2$-PEG nanocomposite films before and after annealed at 50°C/100h.

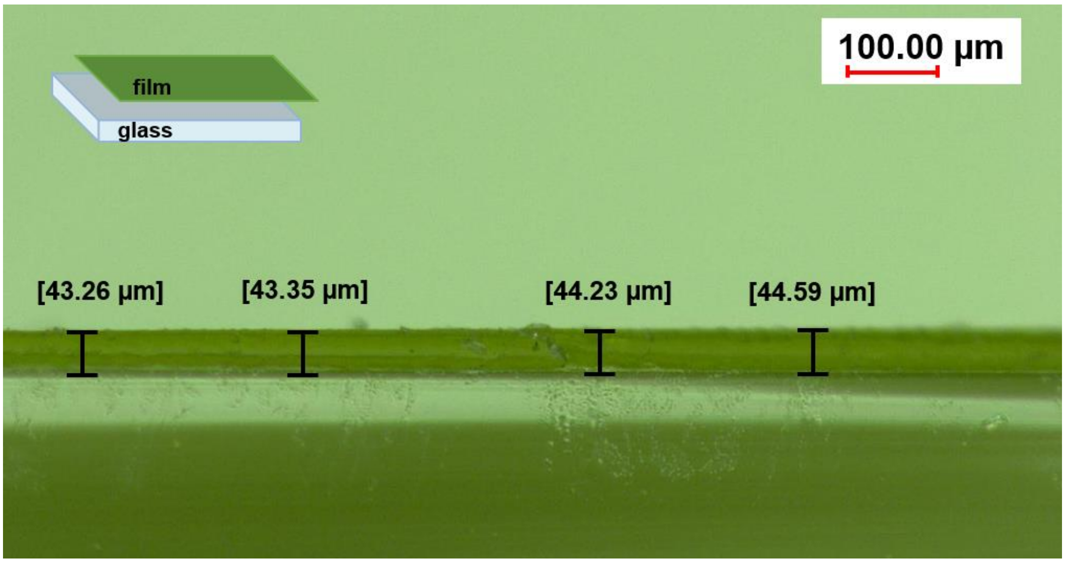

Figure S11. Example of digital microscope pictures of the cross section of a {Nb_5TaBr_{12}}-2@PVP nanocomposite films on glass substrate.

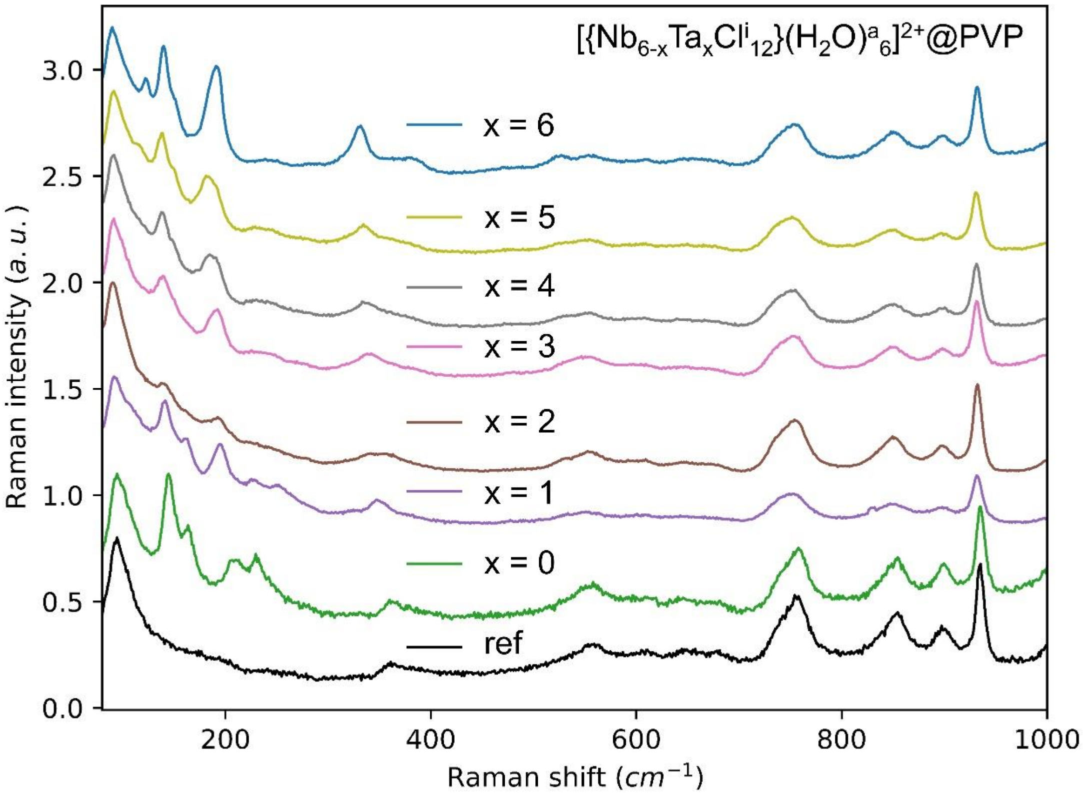

Figure S12. Normalized Raman spectra of the {$Nb_{6-x}Ta_xCl^i_{12}$}-12@PVP nanocomposite films (1 ≤ x ≤ 5]. Ref = @PVP film without metal atom clusters.

Table S3. Main Raman signature of the $\{Nb_{6-x}Ta_xCl^i_{12}\}$-12@PVP nanocomposite films ($0 \leq x \leq 6$. The $\{Nb_6Cl^i_{12}\}$@PVP Raman signature (x = 0) was reported very recently in Ref 1.

**$\{Nb_{6-x}Ta_xCl^i_{12}\}$@PVP**						
x = 0 [1]	360	232 – 213	–	164	144	–
x = 1	347	250 – 227	195	161	142	–
x = 2	343	–	192	160	138	–
x = 3	338	–	192	–	139	–
x = 4	333	–	184	–	138	–
x = 5	334	–	181	–	138	119
x = 6	330	–	188	–	138	122

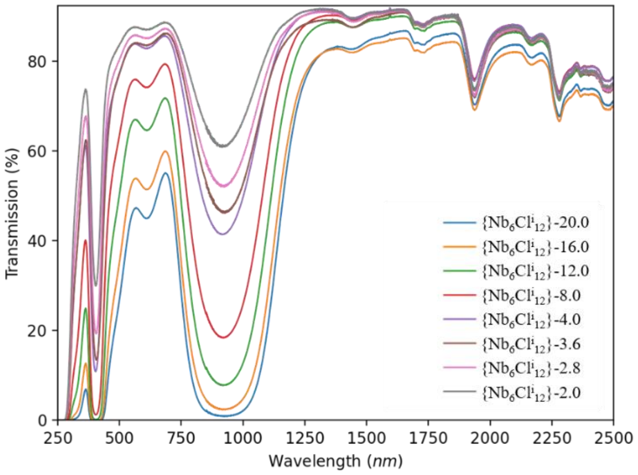

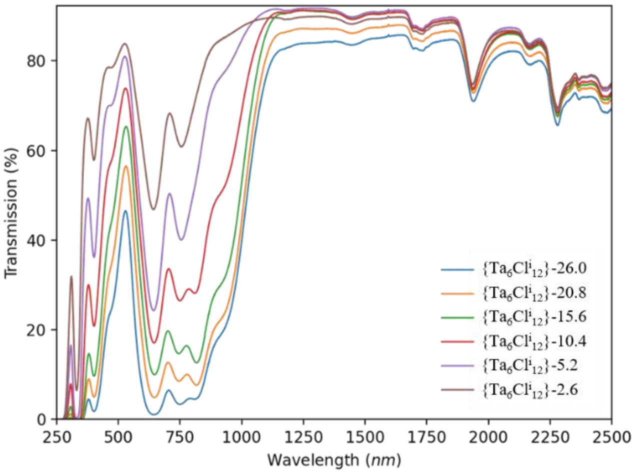

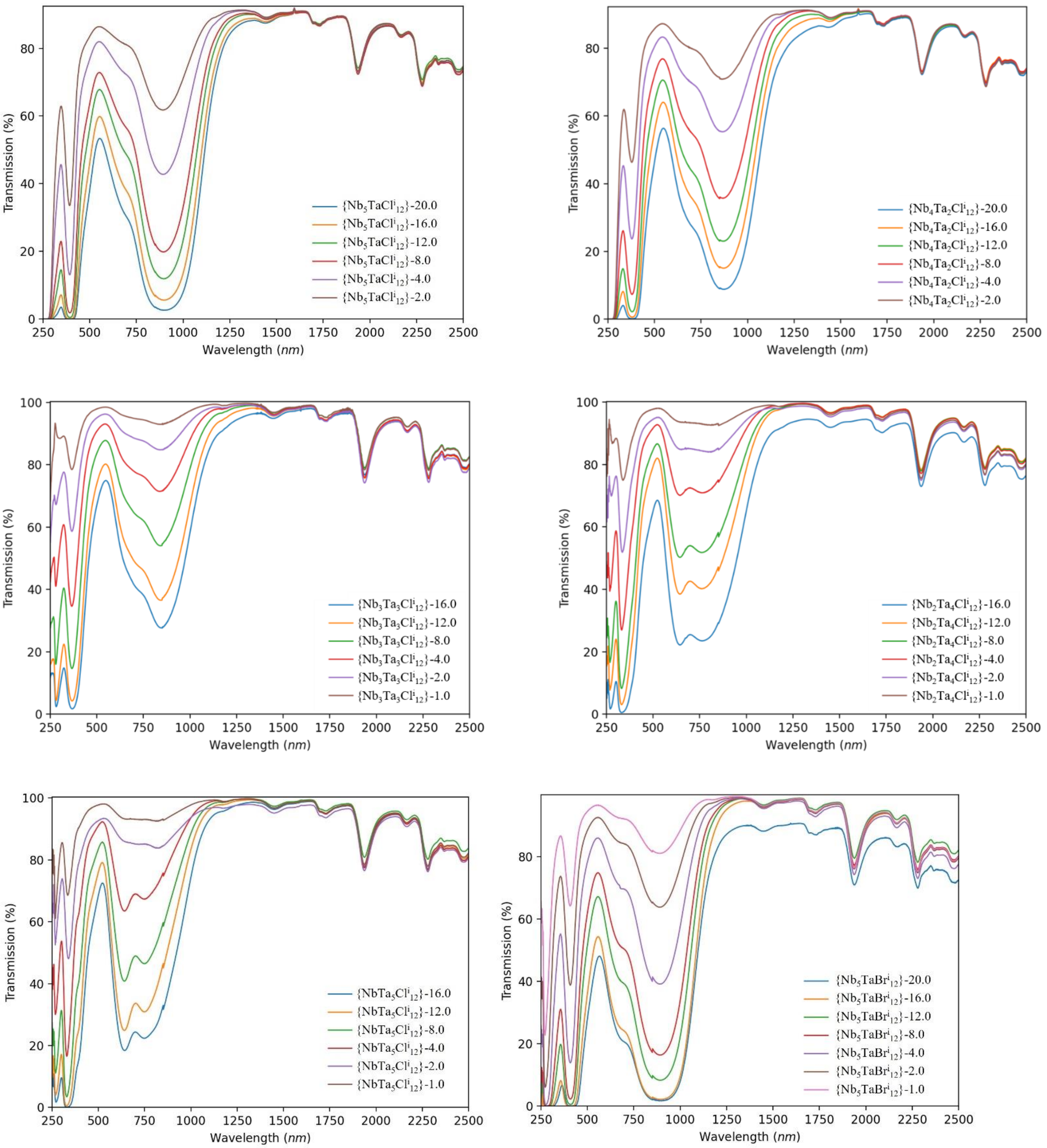
Transmission (%)
Wavelength (nm)
{$Nb_5TaCl^i_{12}$}-20.0
{$Nb_5TaCl^i_{12}$}-16.0
{$Nb_5TaCl^i_{12}$}-12.0
{$Nb_5TaCl^i_{12}$}-8.0
{$Nb_5TaCl^i_{12}$}-4.0
{$Nb_5TaCl^i_{12}$}-2.0
{$Nb_4Ta_2Cl^i_{12}$}-20.0
{$Nb_4Ta_2Cl^i_{12}$}-16.0
{$Nb_4Ta_2Cl^i_{12}$}-12.0
{$Nb_4Ta_2Cl^i_{12}$}-8.0
{$Nb_4Ta_2Cl^i_{12}$}-4.0
{$Nb_4Ta_2Cl^i_{12}$}-2.0
{$Nb_3Ta_3Cl^i_{12}$}-16.0
{$Nb_3Ta_3Cl^i_{12}$}-12.0
{$Nb_3Ta_3Cl^i_{12}$}-8.0
{$Nb_3Ta_3Cl^i_{12}$}-4.0
{$Nb_3Ta_3Cl^i_{12}$}-2.0
{$Nb_3Ta_3Cl^i_{12}$}-1.0
{$Nb_2Ta_4Cl^i_{12}$}-16.0
{$Nb_2Ta_4Cl^i_{12}$}-12.0
{$Nb_2Ta_4Cl^i_{12}$}-8.0
{$Nb_2Ta_4Cl^i_{12}$}-4.0
{$Nb_2Ta_4Cl^i_{12}$}-2.0
{$Nb_2Ta_4Cl^i_{12}$}-1.0
{$NbTa_5Cl^i_{12}$}-16.0
{$NbTa_5Cl^i_{12}$}-12.0
{$NbTa_5Cl^i_{12}$}-8.0
{$NbTa_5Cl^i_{12}$}-4.0
{$NbTa_5Cl^i_{12}$}-2.0
{$NbTa_5Cl^i_{12}$}-1.0
{$Nb_5TaBr^i_{12}$}-20.0
{$Nb_5TaBr^i_{12}$}-16.0
{$Nb_5TaBr^i_{12}$}-12.0
{$Nb_5TaBr^i_{12}$}-8.0
{$Nb_5TaBr^i_{12}$}-4.0
{$Nb_5TaBr^i_{12}$}-2.0
{$Nb_5TaBr^i_{12}$}-1.0

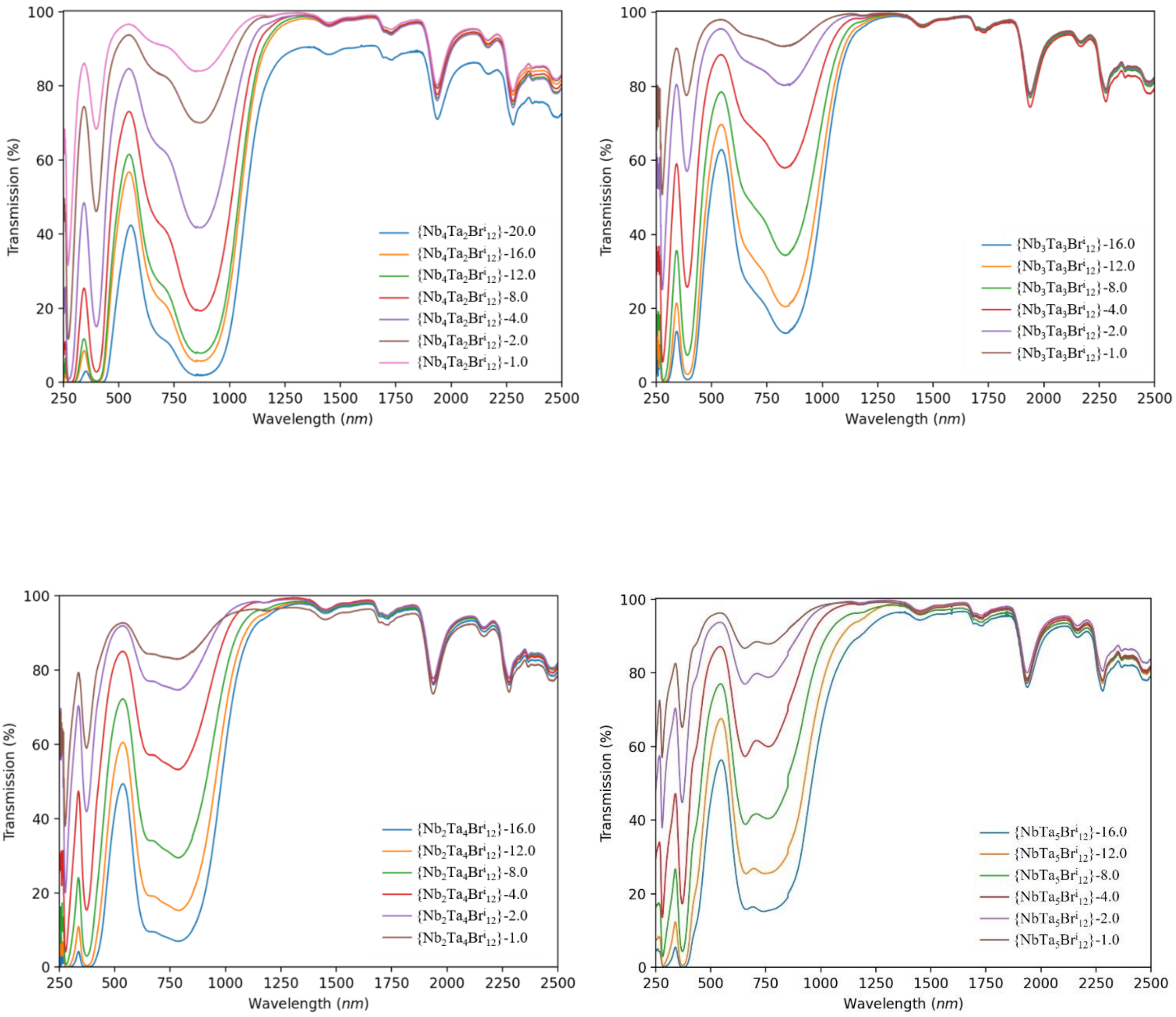

Figure S13. UV-Vis-NIR transmission spectra of the $\{Nb_{6-x}Ta_xX^i_{12}\}$-Y@PVP (X = Cl, Br; $0 \leq x \leq 6$) nanocomposite films for concentrations ranging from 1 to 20 g·L^{-1}.

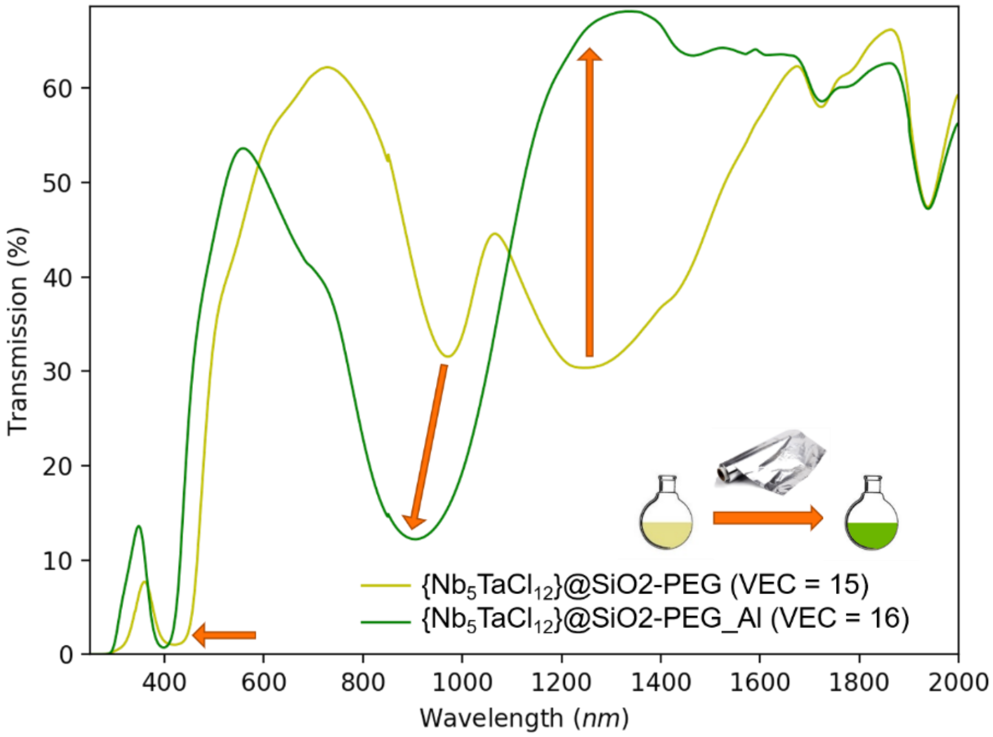

Figure S14. UV-Vis-NIR transmission spectra of the $\{Nb_5TaCl_{12}\}@SiO_2$-PEG nanocomposite films without and with aluminum metal as reducing agent.

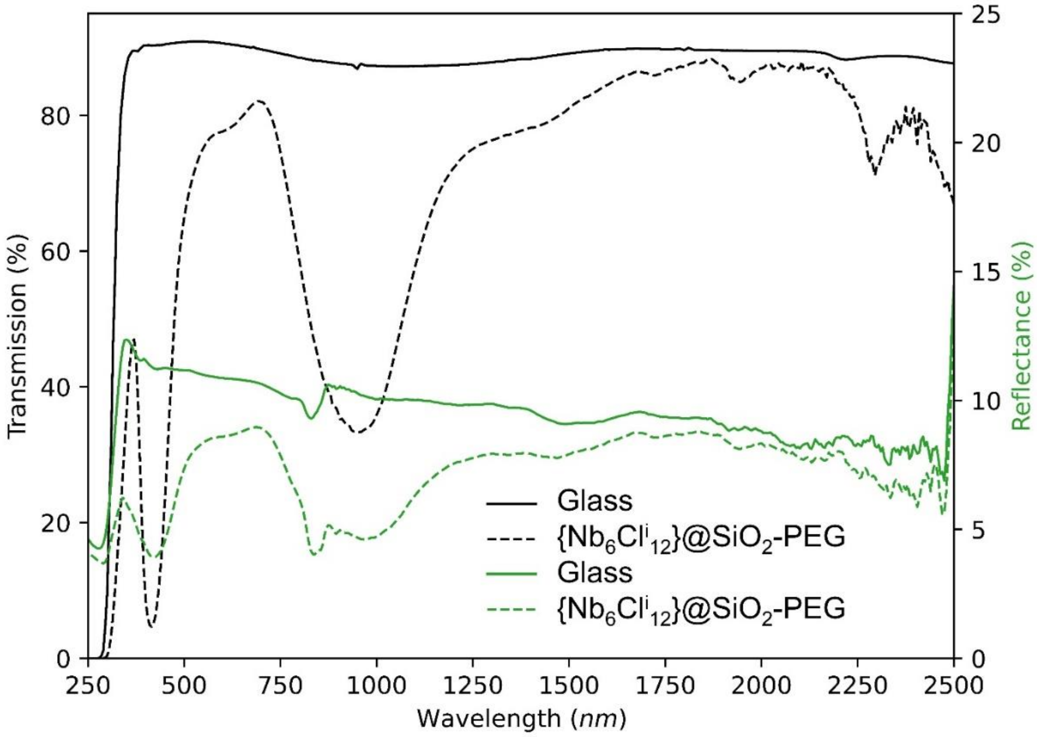

Figure S15. UV-Vis-NIR transmission and reflectance spectra of the glass substrate and the $\{Nb_6Cl_{12}\}@SiO_2$-PEG nanocomposite films.

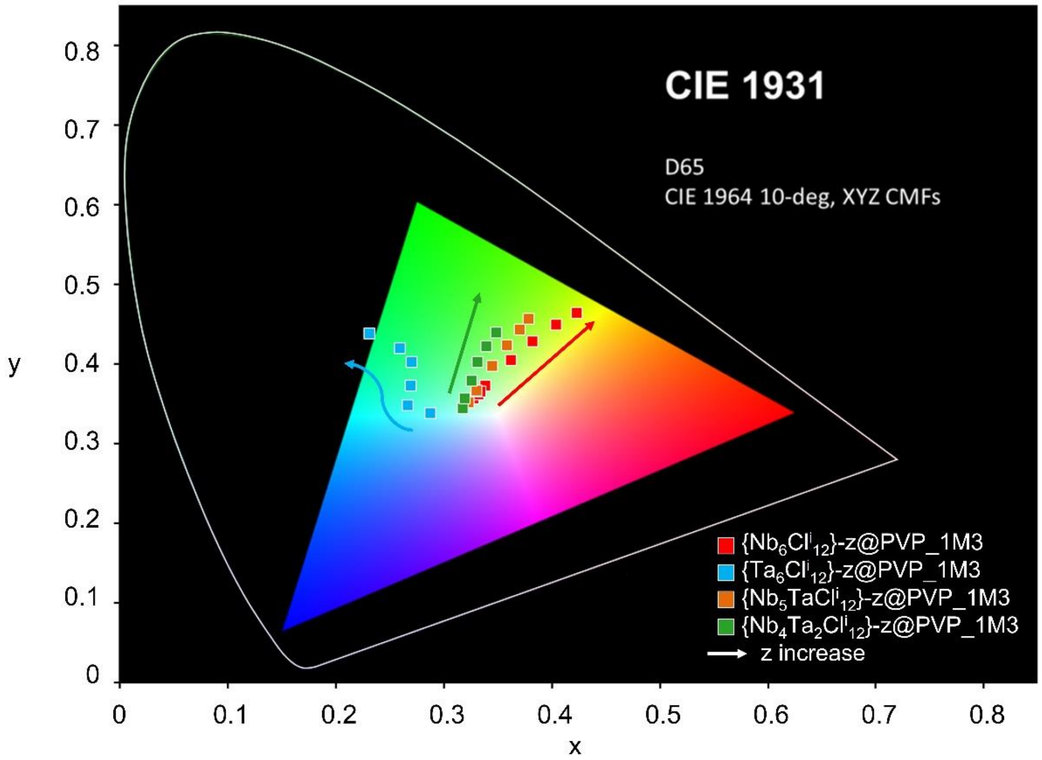

Figure S16. CIE chromaticity coordinates for the nanocomposite films based on chlorine cluster compounds with x = 0, 1, 2 and 6.

Figure-of-merit (FOM) values based on CIE Colorimetric coordinates, T_L (visible transmittance), T_E (solar transmittance), S_{NIR} (NIR shielding), haze and clarity were measured. T_L, T_E and S_{NIR} were obtained using the equations 1, 2 and 3 respectively [2,3].

$$T_L = \frac{\int_{380}^{760} T(\lambda)S(\lambda)Y(\lambda)d\lambda}{\int_{380}^{760} S(\lambda)Y(\lambda)d\lambda} \qquad \text{Equation S1}$$

$$T_E = \frac{\int_{300}^{2500} T(\lambda)S(\lambda)\, d\lambda}{\int_{300}^{2500} S(\lambda)\, d\lambda} \qquad \text{Equation S2}$$

$$S_{NIR} = 100 - \frac{\int_{760}^{2500} T(\lambda)S(\lambda)\, d\lambda}{\int_{760}^{2500} S(\lambda)\, d\lambda} \qquad \text{Equation S3}$$

T_E is the integrated spectral transmittance of a window weighted by the normalized solar energy distribution spectrum, S. S represents the Air Mass 1.5 which is equivalent to the spectrum of solar radiation after passing through 1.5 times the perpendicular atmospheric thickness. T_L is calculated similarly, but weighted by the photopic response of the human eye, Y.

The color coordinates (x, y, z) thanks to the procedure defined by the International Commission on Illumination (CIE 1931). [4–8] Thus, we used the CIE standard illuminant D65, corresponding roughly to the average midday light in Western Europe / Northern Europe and the standard colorimetric observer for the 10° field obtained from the combined measurements of Stiles and Speranskaya.

The spectral transmittance spectrum of the sample T(λ) is multiplied by the spectral power distribution of a reference illuminant I(λ) giving the following equations:

$$X = \frac{1}{N} \int \bar{x}(\lambda) \times T(\lambda) \times I(\lambda)\, d\lambda \qquad \text{Equation S4}$$

$$Y = \frac{1}{N} \int \bar{y}(\lambda) \times T(\lambda) \times I(\lambda)\, d\lambda \qquad \text{Equation S5}$$

$$Z = \frac{1}{N} \int \bar{z}(\lambda) \times T(\lambda) \times I(\lambda)\, d\lambda \qquad \text{Equation S6}$$

$$N = \int \bar{y}(\lambda) \times I(\lambda)\, d\lambda \qquad \text{Equation S7}$$

Where $\bar{x}$, $\bar{y}$ and $\bar{z}$ are the CIE standard observer functions (10 degrees). The integrals are computed over the visible spectrum (from 360 nm to 830 nm). We used the common reference D65. In practice, the functions found in these integrals exist either from empirical experiments or by measurement. Therefore, there are not mathematical equations representing them. Instead, they exist as discrete samples and so the integrals are replaced by summations:

$X = \frac{1}{N} \sum_i \bar{x}_i \times S_i \times I$ Equation S8

$Y = \frac{1}{N} \sum_i \bar{y}_i \times S_i \times I_i$ Equation S9

$Z = \frac{1}{N} \sum_i \bar{z}_i \times S_i \times I_i$ Equation S10

$N = \sum_i \bar{y}_i \times I_i$ Equation S11

Then, given a XYZ colour whose components are in the nominal range [0,1]:

$x = \frac{X}{X+Y+Z}$ Equation S12

$y = \frac{Y}{X+Y+Z}$ Equation S13

$z = \frac{Z}{X+Y+Z}$ Equation S14

The L* a* b* coordinates were obtained by using this conversion equation:

$L^* = 116\, f\left(\frac{Y}{Y_n}\right) - 16$ Equation S15

$a^* = 500\left(f\left(\frac{X}{X_n}\right) - f\left(\frac{Y}{Y_n}\right)\right)$ Equation S16

$b^* = 200\left(f\left(\frac{Y}{Y_n}\right) - f\left(\frac{Z}{Z_n}\right)\right)$ Equation S17

Table S4. FOM for all the SiO_2-PEG films with VEC = 16.

Cluster core - concentration	T_{vis}	T_{sol}	T_{vis}/T_{sol}	S_{NIR}
$\{Nb_6Cl^i_{12}\}^{2+}$@SiO_2-PEG-45μm	74.8	62.1	1.20	39.7
$\{Nb_6Cl^i_{12}\}^{2+}$@SiO_2-PEG-20μm	83.5	74.9	1.11	26.1
$\{Nb_5TaCl^i_{12}\}^{2+}$@SiO_2-PEG-35μm	77.8	65.7	1.19	34.2
$\{Nb_5TaCl^i_{12}\}^{2+}$@SiO_2-PEG-20μm	82.1	72.8	1.13	27.1
$\{Nb_4Ta_2Cl^i_{12}\}^{2+}$@$SiO_2$-PEG-50μm	72.0	58.9	1.22	38.6
$\{Nb_4Ta_2Cl^i_{12}\}^{2+}$@$SiO_2$-PEG-20μm	80.9	71.9	1.12	26.5

Table S5. FOM for all the PVP films with VEC = 16.

Cluster core - concentration	T_{vis}	T_{sol}	T_{vis}/T_{sol}	S_{NIR}
$\{Nb_6Cl^i_{12}\}$-20.0	41.5	33.8	1.23	63.9
$\{Nb_6Cl^i_{12}\}$-16.0	48.6	35.0	1.28	61.7
$\{Nb_6Cl^i_{12}\}$-12.0	62.2	48.3	1.29	53.7
$\{Nb_6Cl^i_{12}\}$-8.0	72.3	57.9	1.25	45.1
$\{Nb_6Cl^i_{12}\}$-4.0	81.9	70.6	1.16	31.8
$\{Nb_6Cl^i_{12}\}$-3.6	82.0	71.5	1.15	30.1
$\{Nb_6Cl^i_{12}\}$-2.8	84.1	74.8	1.12	26.9
$\{Nb_6Cl^i_{12}\}$-2.0	86.4	78.8	1.10	22.6
$\{Ta_6Cl^i_{12}\}$-26.0	26.3	30.5	0.86	48.0
$\{Ta_6Cl^i_{12}\}$-20.8	37.4	36.4	1.03	44.5
$\{Ta_6Cl^i_{12}\}$-15.6	46.6	42.9	1.09	39.3
$\{Ta_6Cl^i_{12}\}$-10.4	55.6	52.7	1.05	30.7
$\{Ta_6Cl^i_{12}\}$-5.2	62.9	63.8	0.98	19.7
$\{Ta_6Cl^i_{12}\}$-2.6	73.2	74.2	0.99	15.5

$\{Nb_5TaCl^i_{12}\}$-20.0	47.1	35.6	1.32	57.8
$\{Nb_5TaCl^i_{12}\}$-16.0	54.1	40.7	1.33	54.4
$\{Nb_5TaCl^i_{12}\}$-12.0	62.9	48.3	1.30	48.8
$\{Nb_5TaCl^i_{12}\}$-8.0	68.8	54.5	1.26	43.7
$\{Nb_5TaCl^i_{12}\}$-4.0	79.6	68.3	1.16	31.1
$\{Nb_5TaCl^i_{12}\}$-2.0	85.1	77.6	1.10	22.2
$\{Nb_4Ta_2Cl^i_{12}\}$-20.0	50.0	39.5	1.26	51.5
$\{Nb_4Ta_2Cl^i_{12}\}$-16.0	58.1	46.2	1.26	46.2
$\{Nb_4Ta_2Cl^i_{12}\}$-12.0	65.1	52.7	1.23	41.1
$\{Nb_4Ta_2Cl^i_{12}\}$-8.0	72.5	61.1	1.19	34.1
$\{Nb_4Ta_2Cl^i_{12}\}$-4.0	80.7	72.3	1.12	24.8
$\{Nb_4Ta_2Cl^i_{12}\}$-2.0	85.8	80.5	1.07	18.1
$\{Nb_3Ta_3Cl^i_{12}\}$-16.0	61.5	50.8	1.21	40.2
$\{Nb_3Ta_3Cl^i_{12}\}$-12.0	67.4	57.0	1.18	34.8
$\{Nb_3Ta_3Cl^i_{12}\}$-8.0	76.6	67.7	1.13	26.4
$\{Nb_3Ta_3Cl^i_{12}\}$-4.0	83.4	76.9	1.08	19.5
$\{Nb_3Ta_3Cl^i_{12}\}$-2.0	87.9	84.0	1.05	14.5
$\{Nb_3Ta_3Cl^i_{12}\}$-1.0	90.9	88.7	1.02	10.9
$\{Nb_2Ta_4Cl^i_{12}\}$-16.0	45.2	43.5	1.04	39.9
$\{Nb_2Ta_4Cl^i_{12}\}$-12.0	60.6	57.1	1.06	29.3
$\{Nb_2Ta_4Cl^i_{12}\}$-8.0	67.9	64.3	1.06	24.8
$\{Nb_2Ta_4Cl^i_{12}\}$-4.0	78.8	75.6	1.04	17.9
$\{Nb_2Ta_4Cl^i_{12}\}$-2.0	85.1	82.6	1.03	14.7
$\{Nb_2Ta_4Cl^i_{12}\}$-1.0	89.7	87.9	1.02	11.4
$\{NbTa_5Cl^i_{12}\}$-16.0	45.6	45.5	1.00	34.8
$\{NbTa_5Cl^i_{12}\}$-12.0	52.6	52.7	1.00	28.6
$\{NbTa_5Cl^i_{12}\}$-8.0	63.7	62.4	1.02	23.1
$\{NbTa_5Cl^i_{12}\}$-4.0	76.3	74.2	1.03	17.3
$\{NbTa_5Cl^i_{12}\}$-2.0	84.3	82.1	1.03	15.0
$\{NbTa_5Cl^i_{12}\}$-1.0	89.7	88.1	1.02	10.9
$\{Nb_5TaBr^i_{12}\}$-20.0	32.7	26.1	1.25	63.7
$\{Nb_5TaBr^i_{12}\}$-16.0	38.7	30.3	1.28	59.6
$\{Nb_5TaBr^i_{12}\}$-12.0	52.7	40.4	1.30	53.1
$\{Nb_5TaBr^i_{12}\}$-8.0	61.4	48.1	1.28	47.6
$\{Nb_5TaBr^i_{12}\}$-4.0	75.0	62.9	1.19	32.5
$\{Nb_5TaBr^i_{12}\}$-2.0	83.5	75.5	1.10	23.8
$\{Nb_5TaBr^i_{12}\}$-1.0	88.4	83.8	1.05	16.3
$\{Nb_4Ta_2Br^i_{12}\}$-20.0	27.0	23.5	1.15	53.9
$\{Nb_4Ta_2Br^i_{12}\}$-16.0	40.6	33.3	1.22	52.0
$\{Nb_4Ta_2Br^i_{12}\}$-12.0	45.5	36.5	1.24	44.3
$\{Nb_4Ta_2Br^i_{12}\}$-8.0	58.6	47.5	1.23	33.1
$\{Nb_4Ta_2Br^i_{12}\}$-4.0	72.7	62.1	1.17	20.2
$\{Nb_4Ta_2Br^i_{12}\}$-2.0	84.4	77.8	1.08	14.6
$\{Nb_3Ta_3Br^i_{12}\}$-16.0	46.3	39.4	1.17	45.0

$\{Nb_3Ta_3Br^i_{12}\}$-12.0	53.9	45.6	1.18	40.8
$\{Nb_3Ta_3Br^i_{12}\}$-8.0	64.4	55.3	1.16	34.0
$\{Nb_3Ta_3Br^i_{12}\}$-4.0	77.1	69.4	1.11	24.1
$\{Nb_3Ta_3Br^i_{12}\}$-2.0	86.5	81.9	1.05	15.4
$\{Nb_3Ta_3Br^i_{12}\}$-1.0	90.0	87.5	1.03	11.5
$\{Nb_2Ta_4Br^i_{12}\}$-16.0	29.9	30.6	0.98	45.4
$\{Nb_2Ta_4Br^i_{12}\}$-12.0	41.6	39.1	1.06	40.0
$\{Nb_2Ta_4Br^i_{12}\}$-8.0	55.0	55.0	1.10	33.0
$\{Nb_2Ta_4Br^i_{12}\}$-4.0	71.1	65.4	1.09	23.2
$\{Nb_2Ta_4Br^i_{12}\}$-2.0	81.4	77.6	1.05	16.3
$\{Nb_2Ta_4Br^i_{12}\}$-1.0	83.7	81.2	1.03	15.5
$\{NbTa_5Br^i_{12}\}$-16.0	38.6	36.2	1.07	42.5
$\{NbTa_5Br^i_{12}\}$-12.0	50.2	45.5	1.10	35.2
$\{NbTa_5Br^i_{12}\}$-8.0	61.3	56.3	1.09	27.1
$\{NbTa_5Br^i_{12}\}$-4.0	73.8	69.3	1.06	18.8
$\{NbTa_5Br^i_{12}\}$-2.0	83.3	80.3	1.04	13.3
$\{NbTa_5Br^i_{12}\}$-1.0	87.4	85.2	1.02	11.6

References

1. Lebastard, C.; Wilmet, W.; Cordier, S.; Comby-Zerbino, C.; MacAleese, L.; Dugourd, P.; Uchikoshi, T.; Dorcet, V.; Amela-Cortes, M.; Renaud, A.; et al. Nanoarchitectonics of glass coatings for near-infrared shielding: From solid-state cluster-based niobium chlorides to the shaping of nanocomposite films. *ACS Appl. Mater. Interfaces* **2022**, *14*, 21116–21130.
2. Smith, G.B.; Deller, C.A.; Swift, P.D.; Gentle, A.; Garrett, P.D.; Fisher, W.K. Nanoparticle-doped polymer foils for use in solar control glazing. *J. Nanoparticle Res.* **2002**, *4*, 157–165.
3. Gao, Q.; Wu, X.; Huang, T. Greatly improved NIR shielding performance of CuS nanocrystals by gallium doping for energy efficient window. *Ceram. Int.* **2021**, *47*, 23827–23833.
4. Stiles, W.S.; Burch, J.M. NPL colour-matching investigation: final report (1958). *Opt. Acta: Int. J. Opt.* **1959**, *6*, 1–26.
5. Speranskaya NI. Determination of spectral color co-ordinates for twenty-seven normal observers. *Opt. Spectroscopy*, **1959**, 7, 424-428. https://www.scopus.com/record/display.uri?eid=2-s2.0-0001307974&origin=inward&txGid=160a89521936d5c60bb09d3e4dc95d35 (accessed on 4 May 2022).
6. Judd DB. *Proc. CIE Symposium on "Advanced Colorimetry"*, CIE Central Bureau, Vienna, Austria, 1993, 107. https://cie.co.at/publications/proceedings-cie-symposium-advanced-colorimetry-8-10-june-1993-vienna-austria (accessed on 4 May 2022).
7. Trezona, P.W. Derivation of the 1964 CIE 10° *XYZ* colour-matching functions and their applicability in photometry. *Color Res. Appl.* **2001**, *26*, 67–75.
8. Lindbloom, B.J. Site gathered the useful color information, **2017**. http://www.Brucelindbloom.com (accessed on 4 May 2022).
